# Atomistic modeling of alternating access of a mitochondrial ADP/ATP membrane transporter with molecular simulations

**DOI:** 10.1371/journal.pone.0181489

**Published:** 2017-07-20

**Authors:** Koichi Tamura, Shigehiko Hayashi

**Affiliations:** Department of Chemistry, Graduate School of Science, Kyoto University, Kyoto, Japan; Hong Kong University of Science and Technology, HONG KONG

## Abstract

The mitochondrial ADP/ATP carrier (AAC) is a membrane transporter that exchanges a cytosolic ADP for a matrix ATP. Atomic structures in an outward-facing (OF) form which binds an ADP from the intermembrane space have been solved by X-ray crystallography, and revealed their unique pseudo three-fold symmetry fold which is qualitatively different from pseudo two-fold symmetry of most transporters of which atomic structures have been solved. However, any atomic-level information on an inward-facing (IF) form, which binds an ATP from the matrix side and is fixed by binding of an inhibitor, bongkrekic acid (BA), is not available, and thus its alternating access mechanism for the transport process is unknown. Here, we report an atomic structure of the IF form predicted by atomic-level molecular dynamics (MD) simulations of the alternating access transition with a recently developed accelerating technique. We successfully obtained a significantly stable IF structure characterized by newly formed well-packed and -organized inter-domain interactions through the accelerated simulations of unprecedentedly large conformational changes of the alternating access without a prior knowledge of the target protein structure. The simulation also shed light on an atomistic mechanism of the strict transport selectivity of adenosine nucleotides over guanosine and inosine ones. Furthermore, the IF structure was shown to bind ATP and BA, and thus revealed their binding mechanisms. The present study proposes a qualitatively novel view of the alternating access of transporters having the unique three-fold symmetry in atomic details and opens the way for rational drug design targeting the transporter in the dynamic functional cycle.

## Introduction

Membrane transporters are ubiquitous integral membrane proteins that mediate transfers of various substrates across the biological membrane. Despite the diversity of transporters, their transport processes are often characterized by a simple model of the alternating access, where transport of the substrate is accomplished in a cycle of conformational transitions of the transporter protein between an outward-facing (OF) form and an inward-facing (IF) one. The large conformational changes of the transporter proteins essentially involved in the alternating access substrate transport make atomistic understanding of the transport mechanism difficult. Moreover, lack of an atomistic view of the conformational changes in the transport cycle hinders efficient drug development despite that the transporters are regarded as attractive targets [1,2].

Recent increasing availability of high resolution X-ray crystallographic structures of the membrane transporters has provided a fruitful atomistic insight into their transport mechanisms [3,4]. Especially, in the case that atomic structures of both OF and IF forms were solved for protein members of several transporter families which share the same fold [3,4], direct atomistic views of the alternating access were obtained through computational structural modeling [5,6] and molecular dynamics (MD) simulations with advanced sampling techniques [7–11].

However, due to difficulty in determining X-ray crystallographic structures of membrane proteins, structural information on membrane transport processes is still limited. The ADP/ATP carrier (AAC) of the mitochondrial carrier family (MCF), the transporter targeted in the present study, exemplifies the limitation. AAC is a 30 kDa integral membrane protein embedded in the inner membrane of mitochondria, and exchanges a cytosolic ADP for a matrix ATP. Atomic OF structures fixed by an inhibitor, carboxyatractyloside (CATR), have been solved by X-ray crystallography [12–14], and have revealed their unique pseudo three-fold symmetric fold which is qualitatively different from pseudo two-fold symmetry of most transporters of which atomic structures have been solved [15]. However, no atomic structure of the IF form of MCF transporters has been solved for more than a decade since the OF structure was determined [12]. Although a conceptual model of symmetric rotary twist was proposed based on the symmetric features of the primary sequence and the OF structure [16], the model remains vague due to lack of an atomic view. On the other hand, a recent NMR relaxation-dispersion experiment revealed asymmetric motions of the protein in ADP and CATR sensitive conformational transition to a conformationally excited state from the OF structure [17]. Due to the lack of the structural information of the IF form, furthermore, molecular mechanism of binding of a well-known inhibitor, bongkrekic acid (BA), which binds to AAC selectively in the IF form [18,19], is not clear.

**Fig 1:**
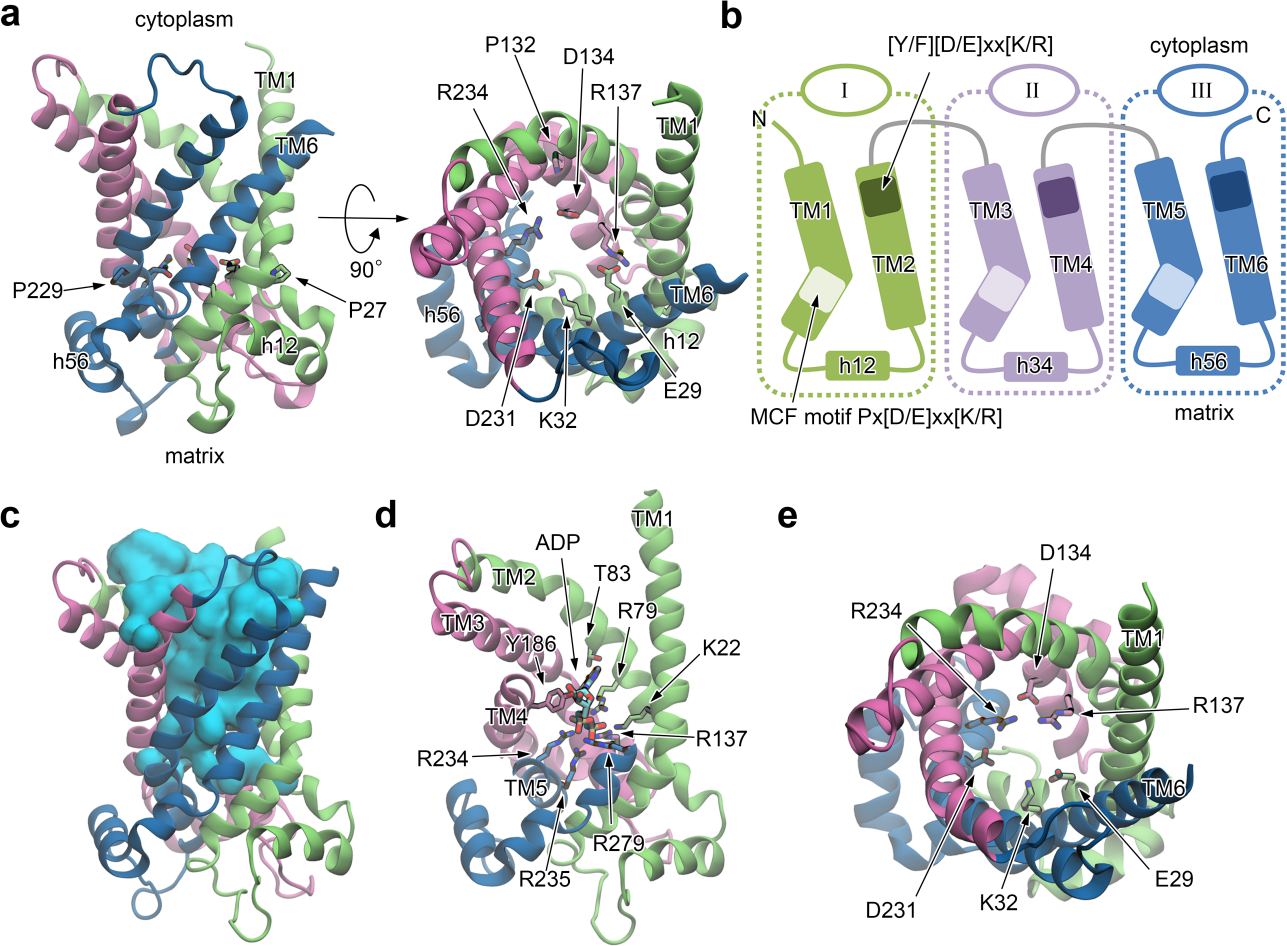
Structure of AAC in the OF form and a binding pose of ADP. (*a*) An X-ray structure of AAC in the OF form (PDB entry 1OKC). The numbering of bovine AAC isoform 1 starts after the initiating methionine (Ser1–), following the numbering in the PDB file. (*b*) Schematic illustration of the protein structure. (*c*) Water distribution in the cytoplasmic pore at the last snapshot of the ADP binding simulation drawn in surface representation and colored in cyan. (*d*) A binding pose of ADP. Residues 190–230 and 280–297 are not shown. (*e*) Charged residues participating in ADP binding from the cytoplasmic side. A view from the cytoplasmic side. ADP is not shown.

In the present study, we predicted an atomic structure of the IF form of AAC from the X-ray crystallographic one of the OF form [12] (Fig 1) by MD simulations with the linear response path following (LRPF) method developed recently [20]. Since the ADP transport of AAC is experimentally known to proceed on a time scale of milliseconds [21], observation of a spontaneous alternating access transition by an unbiased MD simulation is prohibitive. The LRPF method efficiently enhances nonlinear global conformational changes of proteins by iteratively applying biasing forces determined based on the linear response theory and updated in a nonlinear path of conformational changes [22], and enabled us to simulate unprecedentedly large conformational changes of the alternating access at atomic resolution without a prior knowledge of the target protein structure. The IF structure obtained is characterized by newly formed well-packed and -organized inter-domain interactions and was shown to be stable during MD simulations for several microseconds. The simulation also shed light on a mechanism of the strict bidirectional transport selectivity of adenosine nucleotides over guanosine and inosine ones [23–25]. The IF form was then shown to bind ATP and BA and thus revealed their binding mechanisms. The present study proposes a qualitatively novel view of the alternating access of transporters having the unique three-fold symmetry in atomic details and opens the way for rational drug design targeting the transporter in the form of which the atomic structure is experimentally unknown.

## Results

### Structure of OF AAC

An X-ray crystallographic protein structure binding an inhibitor, CATR, represents an OF form accessing to the cytoplasmic side of the inner mitochondrial membrane (Fig 1) [12]. Six transmembrane helices (TM1–6) are arranged pseudo three-fold symmetrical with respect to the membrane normal and form a basket shape opening toward the cytoplasmic side (Fig 1*A*). The structural pseudo three-fold symmetry originates from the tripartite primary sequence. Consequently, the entire protein structure can be divided into three homologous domains, I to III (Fig 1*B*). The channel pore continues from the intermembrane space to the middle of the membrane (Fig 1*C*) as seen in MD simulations of the OF form based on the X-ray crystallographic structure (PDB-ID: 1OKC) [12] in solvent and membrane environment [26,27] (see Materials and Methods). On the other hand, the pore is closed in the matrix side of the channel (Fig 1*C*) for preventing proton leakage across the membrane required for the mitochondrial membrane transporter.

Each odd numbered helix includes a Px[D/E]xx[K/R] sequence called the mitochondrial carrier family (MCF) motif conserved among MCF [28] (Fig 1*B*). The proline residue of the MCF motif (Pro27, Pro132 and Pro229, respectively) induces a large kink in the helix which provides the closed bottom of the basket shape in the matrix half (Fig 1*A*). The charged residues of the MCF motifs at the bottom of the pore (D/E or K/R) extensively forming an inter-domain salt-bridge network (Fig 1*A*) and a cluster of well-conserved hydrophobic residues of odd-numbered helices [29] located immediately beneath the salt-bridge network close the channel in the matrix half.

### OF structure with a bound ADP substrate

We first performed unbiased MD simulations of spontaneous binding of ADP substrate to the OF form of AAC (see Materials and Methods). The substrate reached the layer of the matrix salt-bridge network by ~20 ns (Fig A*a* in S2 Text) and resulted in formation of a binding pose of the carrier (Fig 1*D*). The binding of ADP to the OF form of AAC was also observed in previous MD simulations [25–27]. The protein retained its structural fold during the simulation (Fig 1*C*, 1*D and* 1*E*); the root-mean-square deviation (RMSD) of C_α_ atoms with respect to the X-ray structure during the simulation for ~500 ns after the ADP binding was ~2.5 Å (Fig A*b* in S2 Text), which is comparable with that of a previous study [27]. The small deviation from the X-ray structure is presumably due to the removal of CATR inhibitor in the X-ray structure and the binding of ADP substrate.

The pyrophosphate moiety of ADP formed extensive salt-bridges with six basic residues (Lys22, Arg79, Arg137, Arg234, Arg235, and Arg279). The importance of Lys22, Arg79, and Arg279 in the recognition of ADP was commonly found in previous studies [25–27]. In addition, the present observation suggests that Arg137, Arg234 and Arg235, which are functionally important [29,30] and locate deeper in the cavity, contribute equally to the recognition (Fig 1*D*). The formation of the salt-bridges of the pyrophosphate moiety with the basic residues disrupted the matrix salt-bridge network in the apo structure; the salt-bridges connecting domains I to II and III, i.e., those between Glu29 and Arg137 and between Lys32 and Asp231, respectively, were broken while that between Asp134 and Arg234 connecting domains II and III was preserved, and consequently domain I was isolated (Fig 1*E* and Figs A*c*,*d*,*e* in S2 Text). The isolation of domain I obtained by the present unbiased simulation of the spontaneous binding of ADP for ~500 ns was also observed in one of two models of a previous study with biased steered MD simulations for 20 ns [26]. Disruption of all of the inter-domain salt bridges in the other model of the previous study was not seen in the present unbiased MD simulation. In general, short MD simulations with steering bias could give multiple conformations which are not guaranteed to be equally stable due to high dimensionality of conformational space. The present unbiased MD simulation for 500 ns, which is 25-fold longer than the previous biased steered MD simulations [26], clearly showed that the asymmetric binding of the pyrophosphate moiety seen in both of the previous and present studies is conformationally stable. Conformations of the ribose and adenine moieties underwent large fluctuation as also seen in a previous study [26] and were mostly recognized by highly conserved Tyr186 and Thr83 [29,31], respectively (Fig 1*D*). The recognition of Tyr186 is also consistent with previous studies [25–27]. In the fluctuation of the adenine ring, it also transiently contacted with Ile183, which was experimentally found to be photo-labelled with 2-azido-ATP (ADP) with a minor excess [32,33]. However, given a large size of the azido group, a more elaborative study with azido-ADP would be necessary to precisely examine the binding process underlying the photo-label experiments.

### Structural transition to an ADP-bound IF form

We employed LRPF method [20] in which directed biasing forces are applied to protein atoms to induce functionally relevant large and global conformational changes of the protein during MD simulations. The directed biasing forces were determined based on the linear response theory [22] that defines protein’s global conformational response to local perturbative forces related to its function such as ligand binding and enzymatic reaction. Non-linearity of the conformational changes was then taken into account by an iterative procedure that updates the linear response biasing forces during the MD simulations [20]. A cycle of the iterative procedure also includes a sufficiently long-time (typically tens of nanoseconds) unbiased MD simulation for conformational relaxation to stably follow a non-linear path of conformational changes. The LRPF method was, in fact, shown to be capable of predicting a significantly non-linear closed-to-open conformational transition of the N-terminal domain of calmodulin upon binding of ligand calcium ions [20]. Detailed procedures of the LRPF simulations are described in Materials and Methods and Supporting Information (S1 Text). Although the LRPF method is not designed to determine the exact minimum free energy path of conformational transition and provides an approximate transition path, the high efficiency given by the approximation enables one to predict the final structure of the alternating access undergoing considerably large conformational changes without a target structure [20].

**Fig 2:**
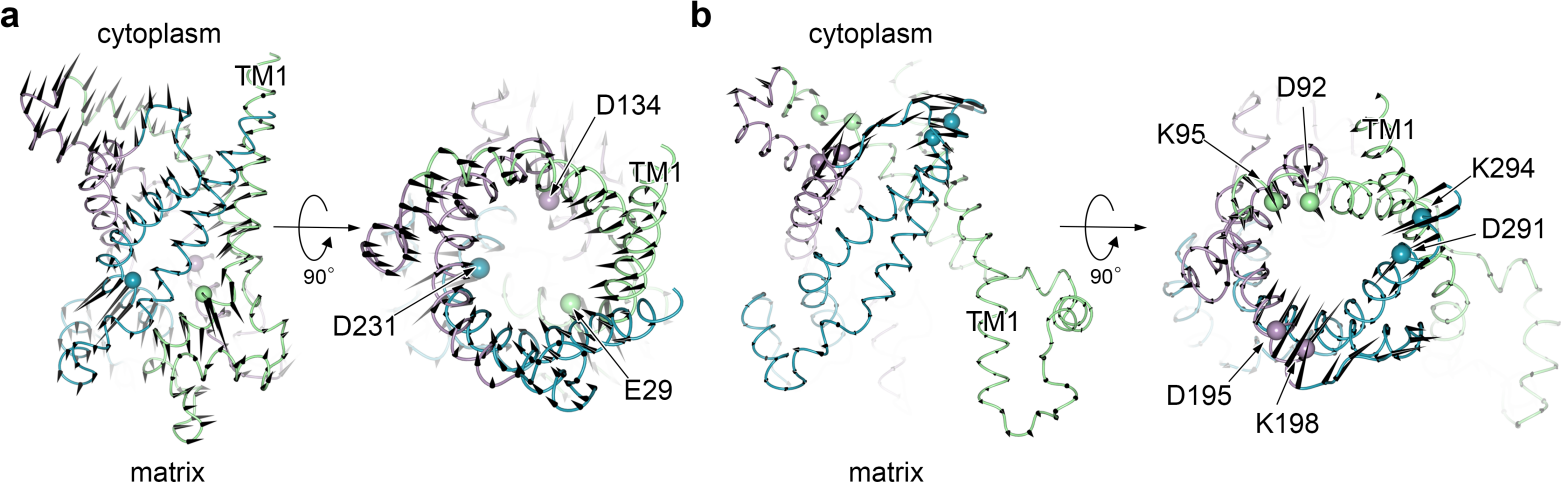
Typical biasing forces used in LRPF simulations. (*a-b*) Typical biasing forces, $F_{matrix}^{bias}$ (*a*) and $F_{cytoplasm}^{bias}$ (*b*), applied to C_α_-atoms in LRPF2 and LRPF2+ simulations, respectively, are represented by black cones. Protein backbone is drawn in ribbon representation. C_α_-atoms to which the perturbative forces, $F_{matrix}^{perturb}$ and $F_{cytoplasm}^{perturb}$, are applied are indicated by spheres. The biasing forces were frequently updated during the LRPF simulations.

In the present simulations of the alternating access transition of AAC, we employed two different perturbative forces, $F_{matrix}^{perturb}$ and $F_{cytoplasm}^{perturb}$, defined based on primary sequence information and structural consideration to open and close the matrix and cytoplasmic channels in two sequential LRPF simulations (see below), respectively. We first chose the acidic amino acids in the well-conserved MCF motifs [28], Px[D/E]xx[K/R], located in the odd numbered helices, i.e., Glu29, Asp134, and Asp231 (Figs 1*A*, 1*B* and 2*A*), for the groups to which the perturbative forces, $F_{matrix}^{perterb}$, were applied. Because the acidic residues are located in the vicinity of the basic residues which bind the pyrophosphate moiety of ADP at the bottom of the pore in the cytoplasmic half, the repulsive forces at the acidic residues originating from the interaction with the pyrophosphate are expected to contribute to opening of the pore in the matrix half upon ADP binding. The first perturbative forces, $F_{matrix}^{perterb}$, were therefore set to be the forces pushing C_α_ atoms of the acidic residues outward (see S1 Text for details). Then, through the linear response theory, the perturbative forces determined the biasing forces which were actually applied to all of C_α_ atoms of the entire protein in the MD simulations to enhance global conformational changes. Four independent LRPF simulations which consisted of 17–43 cycles of update of the biasing forces, respectively, were performed.

Fig 2*A* depicts typical biasing forces, $F_{matrix}^{bias}$, obtained with the perturbative forces, $F_{matrix}^{perterb}$. Because of the outward perturbative forces at the acidic residues, large biasing forces pushing around the acidic residues to open the matrix half of the channel were produced. Furthermore, the global correlation of the protein dynamics taken into account through the linear response procedure provides large biasing forces in the cytoplasmic side of the protein in the directions toward closing the cytoplasmic pore. The strongly correlated biasing forces are of crucial importance to induce the conformational transition to the IF form as described below.

The second perturbative forces, $F_{cytoplasm}^{perturb}$, are applied to charged residues in another well-conserved motifs, [Y/F][D/E]xx[K/R] [16], located in the cytoplasmic side of the even-numbered helices (Fig 1*B*). The charged residues are exposed into the open channel in the cytoplasmic side of the OF form, and clear inter-domain salt-bridges as observed in the matrix half were not observed because of large distances between those groups from the different domains in the OF form. Kunji and co-workers suggested that the charged groups in the cytoplasmic motifs undergo formation of inter-domain salt-bridges upon the conformational transition to an IF form [14,16]. We therefore adopted the second perturbative forces that decrease distances between the charged groups from the different domains to supplement the closure of the channel in the cytoplasmic side (see S1 Text for details). The biasing forces, $F_{cytoplasm}^{bias}$, obtained by the second perturbative forces, $F_{cytoplasm}^{perturb}$, were observed to distribute in the cytoplasmic side in the directions toward closing the cytoplasmic pore (Fig 2*B*).

It should be noted that the perturbative forces, $F_{matrix}^{perturb}$ and $F_{cytoplasm}^{perturb}$, and the biasing forces, $F_{matrix}^{bias}$ and $F_{cytoplasm}^{bias}$, obtained by the linear response theory with the perturbative forces, respectively, were nearly three-fold symmetric because of the three-fold symmetry of the sequential motifs and the protein structure. Asymmetric conformational changes induced in the LRPF simulations are therefore attributed to asymmetric molecular interactions already existing in the molecular structure of the protein-ligand complex.

**Fig 3:**
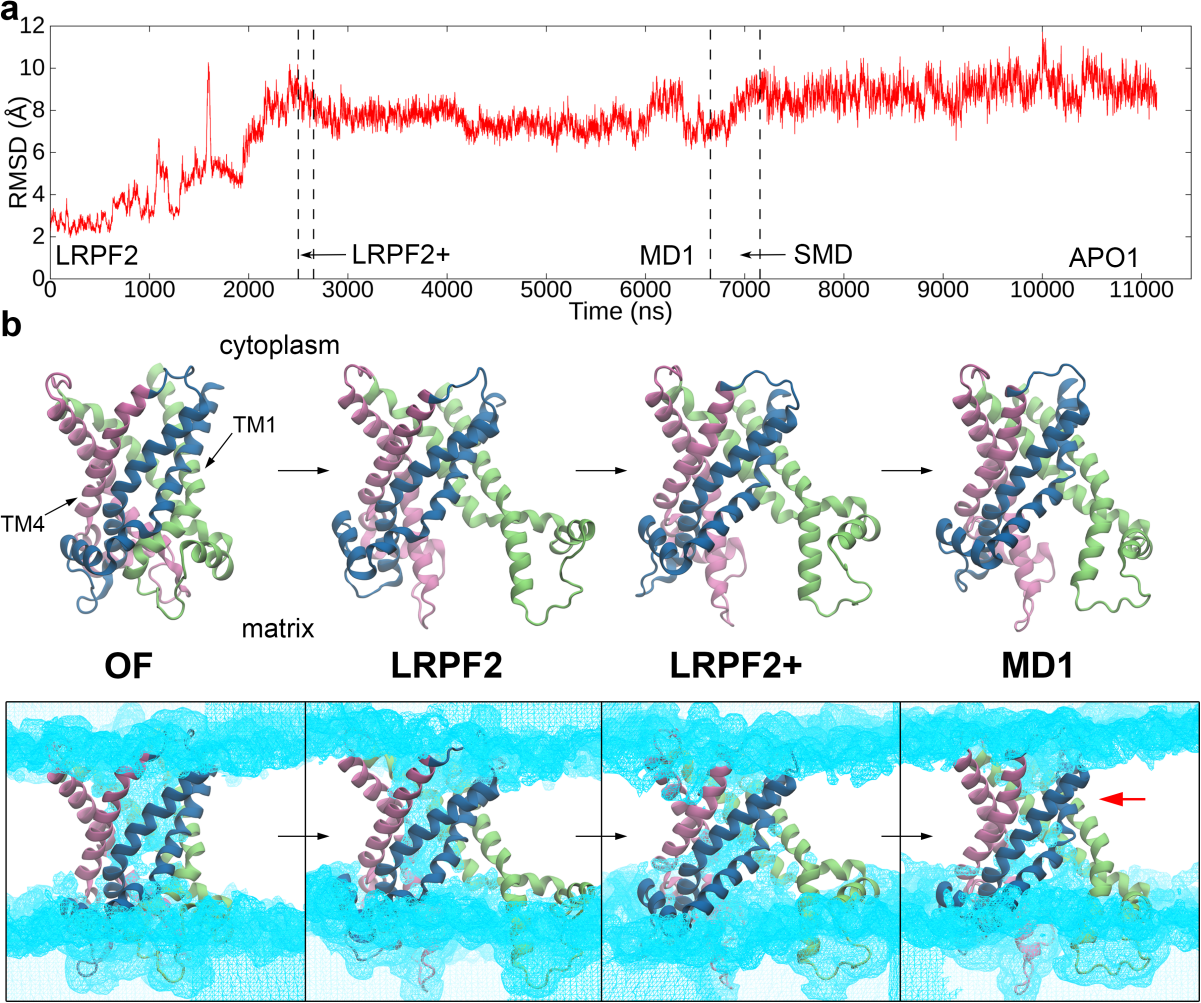
Conformational transition from the OF form to the IF one. (*a*) Time course of C_α_-RMSD with respect to the X-ray structure along LRPF2, LRPF2+, MD1, SMD and APO1 trajectories. In LRPF2 and LRPF2+ simulations, biasing forces of $F_{matrix}^{bias}$ and $F_{cytoplasm}^{bias}$ to stimulate opening and closure of the matrix and cytoplasmic sides of the protein, respectively, were applied. MD1 was an unbiased MD simulation after the LRPF simulations of the forced conformational changes. ADP substrate was then removed by forced transportation to the matrix with a steered MD method in SMD simulation. Finally, the apo protein was equilibrated by unbiased MD simulation in APO1 simulation. Details of the simulation protocols are summarized in Table 1 in S2 Text. (*b*) Conformational changes from the ADP-bound OF form to the ADP bound IF form. The last snapshots of the ADP binding simulation (OF), LRPF2, LRPF2+ and MD1 simulations are shown. In bottom panels, water distributions contoured at 0.3 occupancy level for the corresponding simulations are drawn in cyan. The water occupancy was calculated from the last 1-ns portion of each trajectory. A red arrow indicates the cytoplasmic constriction site in the IF form.

The MD simulation of the conformational transition to the IF form was composed of a series of three different simulations, i.e., LRPF2, LRPF2+, and MD1 (Fig 3 and S1 Movie). Details of their simulation protocols are summarized in Table 1 in S2 Text. Four LRPF simulations with the biasing forces, $F_{matrix}^{bias}$, obtained by the first perturbative forces at the MCF motifs, $F_{matrix}^{perturb}$, (LRPF1–4) were first carried out as mentioned above (Fig 3 and Figs B and C in S2 Text), and opening of the matrix channel was completed in ~2.5 μs in one of the LRPF simulations (LRPF2) (Fig 3 and Fig B in S2 Text). A LRPF simulation with the biasing forces, $F_{cytoplasm}^{bias}$, by the second perturbative forces at the cytoplasmic motifs, $F_{cytoplasm}^{perturb}$, succeeding from LRPF2 was then performed to induce closure of the cytoplasmic pore (LRPF2+), and the complete closure of the cytoplasmic channel was finally observed in a following thermal relaxation by an unbiased MD simulation for 4 μs (MD1) (Fig 3 and Fig B in S2 Text).

**Fig 4:**
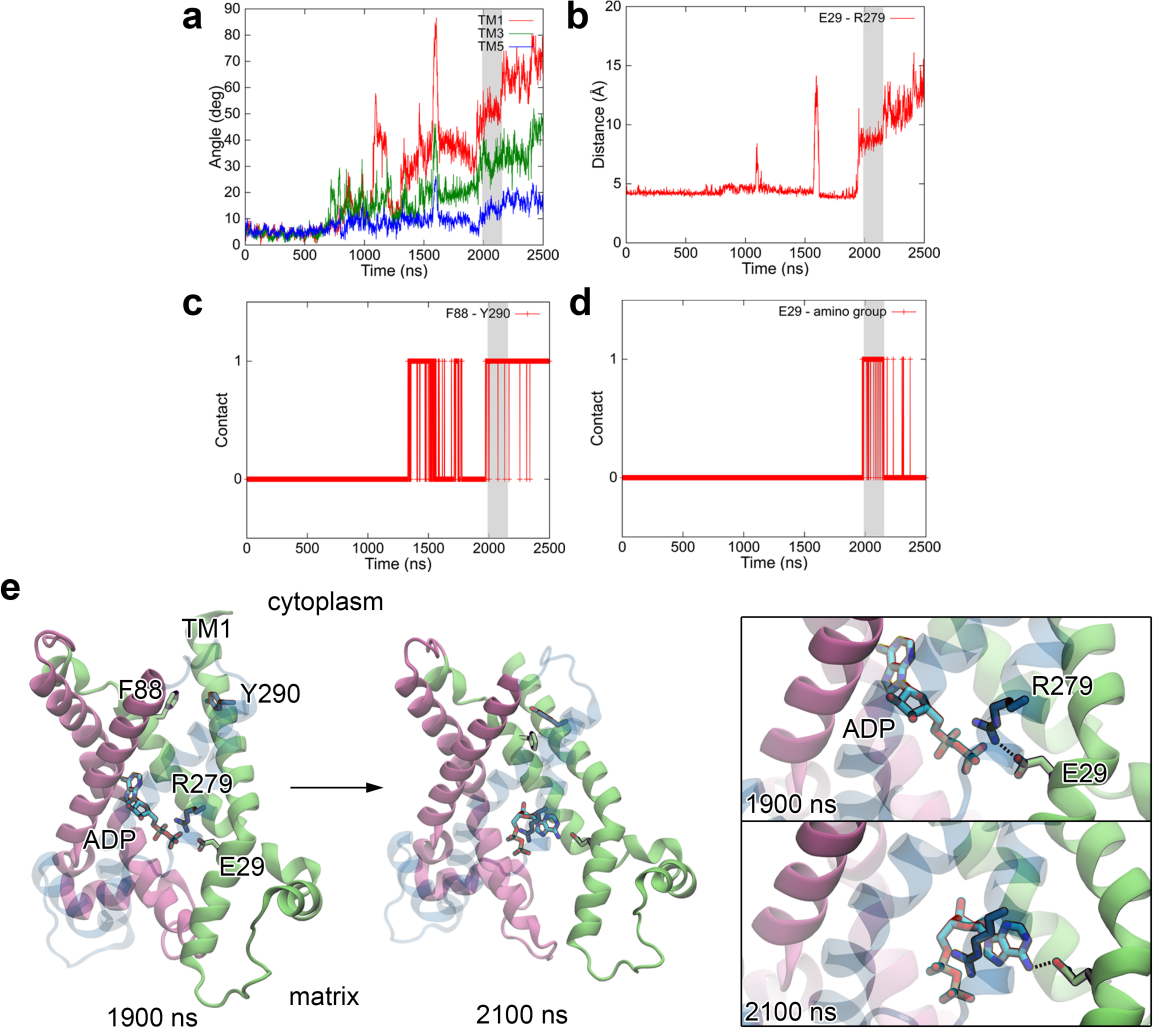
Opening of the matrix side observed in LRPF2 simulation. (*a-d*) Time courses of helix angles (*a*), distance between Glu29 and Arg279 (*b*), contact between Phe88 and Tyr290 (*c*), and contact between Glu29 and the amino group at the adenine ring of ADP (*d*). See Supporting Material for the definition of the helix angle and the contact. Shaded areas indicate that a time region where a hydrogen-bond between Glu29 and the amino group of the adenine ring was established. (*e*) Snapshots of LRPF2 simulation at 1900 (left) ns and 2100 ns (right) before and after formation of the hydrogen-bond between Glu29 and the adenine ring, respectively. Close up views of the ADP binding site are shown in right panels.

The opening of the matrix channel observed in LRPF2 simulation was given by orientational movements of the transmembrane helices accompanied by influx of water molecules into the newly created pore in the matrix side (Figs 3*B* and 4*A* and Figs B*b* and D*a* in S2 Text). TM1 underwent the large orientational movement along with straightening of a strong bend of TM1 at Pro27 resulting in separation of the matrix side of domain-I from the other domains, while the movements of the other symmetry related transmembrane helices, i.e., TM3 and TM5, were relatively moderate (Figs 3*B* and 4*A* and Fig D*a* in S2 Text). The straightening of the bend at a kink at Pro27 which was already seen in the early stage of the opening motion (S1 Movie) is consistent with the experimental evidence of NMR relaxation-dispersion experiment that the strongest chemical exchanges upon ligand sensitive transition to a conformationally excited state asymmetrically appeared in the kink region around Pro27 [17]. The open conformation of the matrix side is also stabilized by newly introduced kinks at Pro82 in TM2 and at the glycine clusters (Gly280, 282, and 283) in TM6 which are sequentially highly conserved [29].

The large orientational movement of TM1 leading to the opening of the matrix channel was accompanied by dissociation and formation of characteristic interactions between residues from different domains, i.e., a salt-bridge between Glu29 and Arg279 in the matrix side and a hydrophobic stacking between Phe88 and Tyr290 in the cytoplasmic side (Fig 4). After the salt-bridge between Glu29 and Arg279 was dissociated at ~2 μs in LRPF2 simulation, the orientational movements of the transmembrane helices developed rapidly, and consequently the matrix channel was opened more widely (Fig 4A, 4*B* and 4*E*). This event was also correlated with the formation of the hydrophobic stacking between Phe88 and Tyr290 in the cytoplasmic side coupled to a tilt of the cytoplasmic side of TM2 in a direction toward closing the cytoplasmic pore, which was enabled by the large orientational movement of TM1 (Fig 4*C* and 4*E*). Note that either of the formation of the salt-bridge or the formation of hydrophobic stacking was not completely established in the other LRPF simulations (LRPF1, 3, and 4) where the opening of the matrix channel was not completed as also seen in the trajectory of LRPF2 before ~2 μs (Fig 4*A* and Fig C in S2 Text). Those interactions therefore play a crucial role in the opening of the matrix channel through the large orientational movement of TM1.

Interestingly, the bound substrate ADP was observed to participate in the conformational transition associated with the opening of the matrix channel. Immediately after the dissociation of Glu29 and Arg279 at ~2 μs in LRPF2 simulation (Fig 4*B*), the adenine ring of ADP moved into a cavity created by the orientational movement of TM1 and its amino group at the position 6 formed a hydrogen-bond with the carboxyl group of the Glu29 for ~200 ns (Fig 4*D* and 4*E*). Because the occupation of the adenine ring into the newly created cavity and the hydrogen-bond of the adenine ring with Glu29 are expected to stabilize the oriented conformation of TM1 and the unstable carboxyl group of Glu29 generated by the dissociation of the salt-bridge with Arg279, respectively, the adenine ring is suggested to contribute to enhancement of the conformational transition correlated with the dissociation of the key salt-bridge. It is noteworthy that the ADP-enhanced conformational transition may partly explain ADP-dependent increase of mobility in TM1 observed in a NMR relaxation dispersion experiment [17].

In the LRPF simulations with $F_{matrix}^{bias}$, LRPF1, LRPF3, and LRPF4, other than LRPF2, the full opening of the matrix side was not observed in the LRPF search time of >4 μs each (Fig C in S2 Text). During the unsuccessful simulations, transient opening of the matrix side leading to partial wetting of the interfaces of three domains in the matrix side were frequently observed. However, the opening events did not result in the full opening of the matrix side characterized by the complete and persistent dissociation of the salt-bridge between Glu29 and Arg279, although the salt-bridge dissociated transiently upon the opening movements of the matrix side. A marked difference between the successful opening of LRPF2 and the others arose in the hydrophobic interaction between Phe88 and Tyr290 in the cytoplasmic side correlated with the opening of the matrix side through the tilt of TM1 as mentioned above (Figs B and C in S2 Text); the correlated hydrophobic stacking between Phe88 and Tyr290 was observed in LRPF2, while such a stacking event was observed in none of the others. The full opening of the matrix side accompanied by the dissociation of the salt-bridge therefore needs the stabilization by the hydrophobic stacking at the remote site in the cytoplasmic side.

It is noteworthy that the hydrophobic stacking was already observed in 1,300–1,800 ns even before the salt-bridge was completely and persistently dissociated later than 2,000 ns (Fig B in S2 Text). The observation indicates that the biasing forces, $F_{matrix}^{bias}$, employed in the LRPF simulations were able to represent, at least partially, the extensive conformational coupling with the remote site leading to the full opening of the matrix side, although they were not well described enough to lead to the full opening in the all of the LRPF searches. More elaborate choices of the perturbative forces and/or protocols of the biasing simulations would be necessary to improve the successful ratio, although they are not easy tasks for the complex and large conformational changes.

Although the large orientational movement of TM1 narrowed the cytoplasmic pore in LRPF2 simulation as mentioned before, the full closure of the channel in the cytoplasmic side was not observed (Fig 3*B*). We therefore performed LRPF2+ simulation with the biasing forces by the second perturbative forces at the cytoplasmic motifs (Fig 3 and Fig D*b* in S2 Text) as mentioned above. In a relatively short time of the LRPF simulation (Fig 3*A*), a tilt of the cytoplasmic side of TM4 in a direction toward closing the cytoplasmic channel was observed (Fig 3*B* and Fig D*b* in S2 Text). The tilt of TM4 was induced by a newly introduced kink at a highly conserved glycine at the position of 182. Upon the movement of TM4, the connectivity of water chains in the cytoplasmic pore was broken (Fig 3*B*), and thus the cytoplasmic channel was almost closed.

The closure of the cytoplasmic channel was fulfilled by formations of inter-domain hydrophobic packing and salt-bridge network (Fig D*b* in S2 Text). In addition to the hydrophobic stacking between Phe88 and Tyr290 observed in LRPF2 simulation, two hydrophobic contacts between Lys91 and Phe191 and between Tyr194 and Leu287, respectively, were newly formed. The inter-domain hydrophobic contacts are highly symmetric; Lys91, Tyr194, and Tyr290 are located at the positions of [Y/F] in the cytoplasmic [Y/F][D/E]xx[K/R] motifs in the even-numbered helices, respectively (Fig 1*B*), and established the hydrophobic contacts with the residues positioned three amino acids apart from [Y/F] of the cytoplasmic motifs toward the N-terminus, i.e., Phe191, Leu287, and Phe88 known as “triplet 89” [34], in the inter-domain partner helices, respectively. Those amino acids constituting the inter-domain hydrophobic packing were known to be sequentially well-conserved; Lys91, Tyr194, and Tyr290 are in the cytoplasmic motifs [16], Phe191 and Tyr194 are part of the so-called “aromatic ladder” [31], and Phe88 and Leu287 are highly preserved among AAC orthologues [29]. Two of the previously proposed inter-domain salt-bridges between the charged groups in the cytoplasmic motifs [14,16], i.e., the salt-bridges between Lys95 and Asp195 and between Lys198 and Asp291, respectively, were also observed, while one of the expected ones, i.e., the salt-bridge between Asp92 and Lys294, was not yet formed (Fig D*b* in S2 Text).

**Fig 5:**
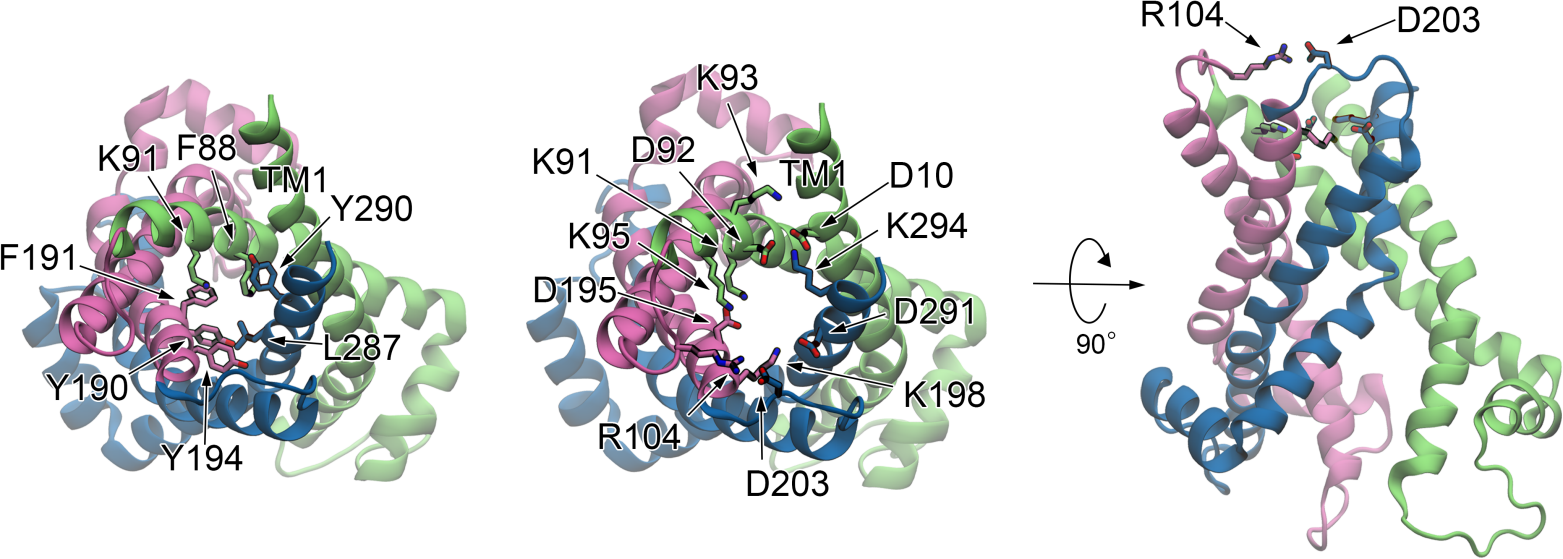
Protein structure of the ADP-bound IF form. Snapshots at 3,990 ns in MD1 simulation are shown. Hydrophobic residues and charged ones participating in the cytoplasmic inter-domain hydrophobic packing and salt-bridge network in the IF form are shown in views from the cytoplasmic side in left and middle panels, respectively. A side view of the protein structure and the cytoplasmic salt-bridge network is shown in a right panel.

The inter-domain hydrophobic packing and salt-bridges in the cytoplasmic side were completed and strengthened in the following conformational relaxation by the unbiased simulation, MD1 (Figs 3 and 5). After a slight tilt of TM4 in the direction toward closing the channel observed at ~50 ns in MD1 simulation, Tyr190, a conserved amino acid from the “aromatic ladder” [31], also participated in the hydrophobic packing (Fig 5). As a result, water molecules were completely excluded in the cytoplasmic side (Fig 3*B*). Furthermore, the last inter-domain salt-bridge between the charged groups in the cytoplasmic motifs, i.e., the one between Asp92 and Lys294, was formed at ~2,500 ns in MD1 simulation (Fig 5). Simultaneously, Asp10 and Lys91 supplemented the inter-domain salt-bridges between the charged groups in the cytoplasmic motifs and a salt-bridge between Arg104 and Asp203 bound the cytoplasmic loops (Fig 5). The charged groups at the cytoplasmic loops are sequentially well conserved in mammals, although they are not across all species. The salt-bridge linkage at the loop is therefore considered to supplementarily contribute to the cytoplasmic closure. The formation of the extensive salt-bridge network in the cytoplasmic side firmly locked the cytoplasmic gate, and completed the full closure of the cytoplasmic channel of the ADP-bound IF form.

The closure of the cytoplasmic channel is requisite for the stable formation of the ADP-bound IF form. We observed that the transmembrane helixes were falling apart in an unbiased MD simulation for 3 μs starting with an initial structure taken from the last snapshot of LRPF2 where the matrix channel was opened while the cytoplasmic channel was not fully closed (Fig 3*B* and Figs D*a* and *c* in S2 Text). The observation therefore suggests that the conformational changes of the opening of the matrix channel and the closure of the cytoplasmic channel revealed in the present simulations need to be tightly coupled, and thus are expected to take place almost simultaneously (on a time scale of less than ~1 μs) in the spontaneous conformational transition process or through an occlude state, where both of the cytoplasmic and matrix channels are closed, if no artificial acceleration is imposed. The tight coupling of the matrix opening and the cytoplasmic closure well explains the blockage of proton permeation necessary for the mitochondrial membrane transporter.

Although those conformational changes appeared in the different LRPF simulations with the biasing forces which provided an approximate transition path, their tight coupling is supported by the relatively short time of LRPF2+ simulation required to induce the inter-domain hydrophobic packing in the cytoplasmic side (Fig 3*A* and Fig D*b* in S2 Text). Several attempts were also made to model an occlude state. For example, targeted MD simulations to induce the characteristic inward movement of TM4 to close the cytoplasmic channel found in the LRPF2+ and MD1 simulations described above were performed without the opening of the matrix side. However, unfortunately, none of the attempts succeeded in obtaining a stable occlude state; closed conformations in the cytoplasmic side induced in those biased simulations were spontaneously relaxed into the open conformation in the initial state when the biasing forces were turned off. Given that the opening of the matrix side was tightly coupled with the hydrophobic stacking between Phe88 and Tyr290 in the cytoplasmic side as described above, formation of an occlude state, if it exists, may also need to be accompanied by conformational changes in the matrix side. More extensive search of conformational changes would be necessary to find a occlude state.

### Structure of an Apo IF form

An IF form of AAC without the substrate ADP was modeled from the ADP-bound IF structure described above. The bound ADP molecule was first dissociated by a steered MD (SMD) simulation for 500 ns from the last snapshot of MD1 simulation (see S1 Text), and removed from the MD system. An unbiased MD simulation for 4 μs (APO1) was then performed to relax the apo IF structure and to assess its structural stability (S2 Movie).

**Fig 6:**
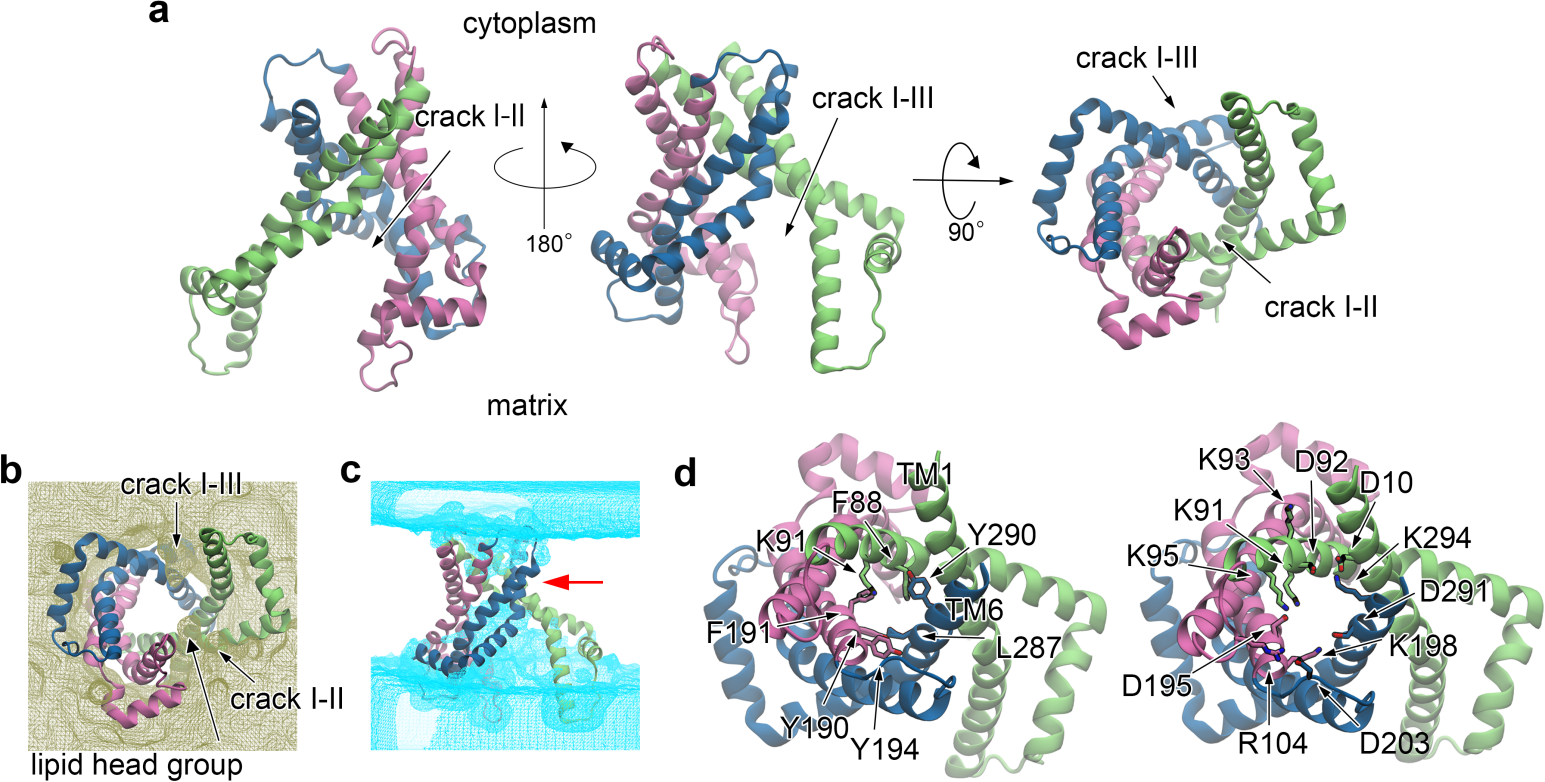
Protein structure in the apo IF form. Results from APO1 simulation were shown. (*a*) The average structure calculated from the last 100-ns portion of the trajectory. (*b-c*) Distributions of lipid molecules (*b*) and water molecules (*c*) contoured at 0.3 occupancy level calculated from the last 100-ns portion of the trajectory are shown. A red arrow indicates the cytoplasmic constriction site in the IF form. (*d*) Hydrophobic residues and charged ones participating in the cytoplasmic inter-domain hydrophobic packing and salt-bridge network in the IF form are shown in views from the cytoplasmic side in left and right panels, respectively.

The dissociation of ADP opened the matrix pore of the IF form more widely, although the overall fold of the ADP-bound IF structure was unchanged (Fig 6*A and* 6*B* and Fig B*a* in S2 Text). RMSD with respect to the OF form and the number of water molecules in the matrix side of the protein slightly increased from those of the ADP-bound IF form, respectively (Fig 3*A* and Fig B*b* in S2 Text). The further opening of the matrix side of the IF form upon the dissociation of ADP is considered to mainly originate from removal of the inter-helix binding through the salt-bridges of the basic residues in the helices with the pyrophosphate of ADP. Thermal fluctuation of the domains was therefore increased after the dissociation of ADP (Fig B*a* in S2 Text). The opening is characterized by the more profound isolation of domain-I, which generated two large clefts between domain-I and the others in the matrix side (Fig 6*A*). A lipid molecule was observed to accommodate in each cleft (Fig 6*B*). Such large clefts are also often found in other membrane transporters of the major facilitator superfamily and ATP-binding cassette family [35–37] as well as in other membrane proteins [38–41]. Transient separation between domain-II and domain III was also observed, although its extent is relatively moderate compared to those of domain-I.

Contrary to the large movements of the protein in the matrix side, the tightly packed structure in the cytoplasmic side which excluded water molecules in the cytoplasmic half of the channel was stably maintained (Fig 6*C*). The inter-domain hydrophobic packing and salt-bridge network formed in the ADP-bound IF form were kept unchanged in APO1 simulation for 4 μs (Fig 6*D*). We also examined structural stability and robustness of the apo IF form by performing three other unbiased MD simulations for 2–3 μs with different initial structures (APO2–4, see Figs E and F in S2 Text). The simulations showed the opening of the matrix side similar to that observed in APO1 and the stably unchanged structures of the cytoplasmic side, indicating the high structural stability and robustness of the apo IF form obtained by the present simulations.

We also performed an MD simulation with an elongated cutoff distance (12 Å) starting from the last snapshot of APO1 simulation. The 2 μs-long simulation (APO1+) further confirmed the structural stability of the apo IF model (Fig G in S2 Text).

### Binding of ATP substrate to the Apo IF form

The IF model was tested if it is capable of binding ATP substrate prerequisite for the substrate transport from the matrix side to the cytoplasm one. For this purpose, eight independent binding simulations (ATP1–8) without any force bias were performed as described in S1 Text. We observed spontaneous binding of ATP to the IF form in all of the binding simulations (Fig H in S2 Text). In the simulations, the triphosphate moiety of ATP was strongly attracted by the basic arginine and lysine residues in the matrix pore of the protein and formed salt-bridges with them. Adenine and ribose moiety of ATP, on the other hand, made contacts with non-polar residues. We did not observe single definite binding pose in the simulations presumably due to limited simulation time (20 ns each). However, some of the basic residues were found to commonly bind to ATP (Fig H in S2 Text). These residues, i.e., Arg235 and Arg279, are known to be functionally important for efficient transport of the substrate [29].

### Binding of bongkrekic acid to the Apo IF form

**Fig 7:**
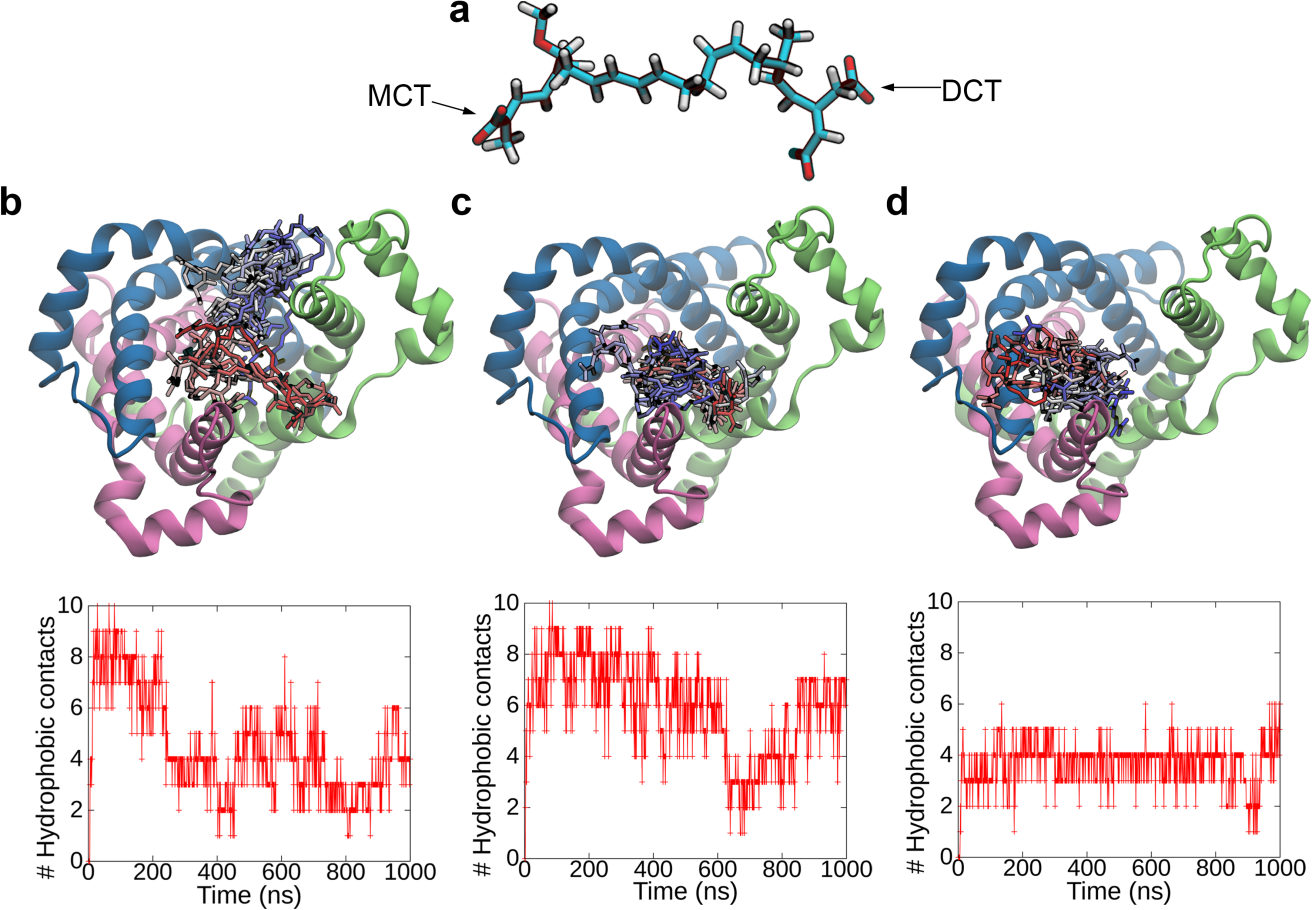
Binding of an inhibitor, BA, to the matrix pore of the apo IF form. (*a*) Structure of BA. Mono-carboxyl terminus (MCT) and di-carboxyl one (DCT) at the two ends of a hydrocarbon chain, respectively, are indicated. (*b-d*) Trajectories of BA in the simulations, BA8 (*b*), BA16 (*c*), and BA20 (*d*). In upper panels, trajectories of BA are mapped to the average structure of the last 100-ns portion of APO3 trajectory. Snapshots of BA at every 10 ns are shown in stick representation. Color is changing from red to white and white to blue along the time course. In lower panels, time courses of hydrophobic contacts are shown. See S1 Text for the definition of the hydrophobic contact.

We performed unbiased MD simulations of spontaneous binding of a bongkrekic acid (BA) which was experimentally known to bind to an IF form of AAC [18,19]. BA consists of a hydrophobic hydrocarbon chain with a methoxy group connecting one carboxylate at one terminus (mono-carboxyl terminus, MCT) and two carboxylates at the other (di-carboxyl terminus, DCT) (Fig 7*A*).

For the binding of BA to the IF form, we first carried out 24 unbiased MD simulations for 20 ns with the apo IF structure obtained by APO3 simulation and one BA placed in different orientations and at different distances to the protein (BA1–24, see S1 Text). We also performed 8 unbiased MD simulations of BA binding for 20 ns with the apo OF structure (BA25–32) for comparison.

We observed binding of BA to the matrix pore in the IF form in 15 simulations out of the 24 ones (Fig I in S2 Text), whereas binding of BA to the cytoplasmic pore in the OF form was not found in any of the 8 simulations (Fig J in S2 Text). The observed stark contrast between the OF form and the IF one regarding the preference of the BA binding is consistent with the experimental evidence that BA binds to the IF form [18,19]. The observed binding structures of the IF form were characterized by binding poses, and accordingly classified into 4 classes (G1–4, Fig G in S2 Text).

The unbiased BA binding simulations of BA8, BA16, and BA20 from G1, G2, and G3 groups, respectively, of which the numbers of salt-bridges and hydrophobic contacts were relatively rich, were then continued until 1 μs to examine the ligand dynamics in the binding processes. In all of the binding processes, BA was continuously attached to the surface of the inside of the matrix pore; BA extensively and continuously established hydrophobic contacts with C-terminal region of odd-numbered helices (Fig 7*B–*7*D*). Furthermore, domain-I, which was separated widely from the other domains and thus exhibited high flexibility, transiently bound to the hydrophobic chain of BA and sandwiched the inhibitor by the hydrophobic interactions (S3–S5 Movies). Since BA underwent slow diffusion on the surface accompanied by repeated formation and dissociation of salt-bridges presumably due to high flexibility of the hydrophobic chain of BA and the opened matrix domains of the protein in the IF form (S3–S5 Movies), clear binding poses were not observed within the simulation time (Fig 7*B–*7*D*). Longer simulations would be required to clarify the final binding pose.

## Discussion

The present LRPF MD simulations for ~10 μs succeeded in predicting an atomic structure of the IF form through the accelerated simulation of the global conformational changes from the OF form to the IF one with RMSD of ~9 Å (Fig 3*A*), which is unprecedentedly large as that obtained by the atomic-level MD simulations without a target structure. The prediction was successfully achieved by the simulation of which the time scale is more than two orders of magnitude shorter than that of ADP transport, i.e., milliseconds, measured experimentally [21]. Even after the large conformational changes from the OF form, the predicted IF structure was found to be well-organized and very stable in MD simulations for 3–4 μs, which is completely different from expected behavior of unfolded proteins. The high stability of the predicted IF structure originates from the precisely well-packed structure of the closed cytoplasmic channel. In fact, upon the formation of the IF form, a radius of gyration of the cytoplasmic channel introduced in a recent study by Pietropaolo et al. [42] to characterize the closure of the channel drastically decreased from ~13 Å to ~9 Å (Fig K in S2 Text), the latter of which is substantially smaller than that of the IF model by Pietropaolo et al. (~12 Å). The newly formed highly packed structure in the cytoplasmic half represented by the significantly small radius of gyration completely excluded the water molecules in the cytoplasmic half and maintained the closed channel conformation very rigid as expressed by small fluctuation of the radius of gyration (Fig K*d* in S2 Text).

**Fig 8:**
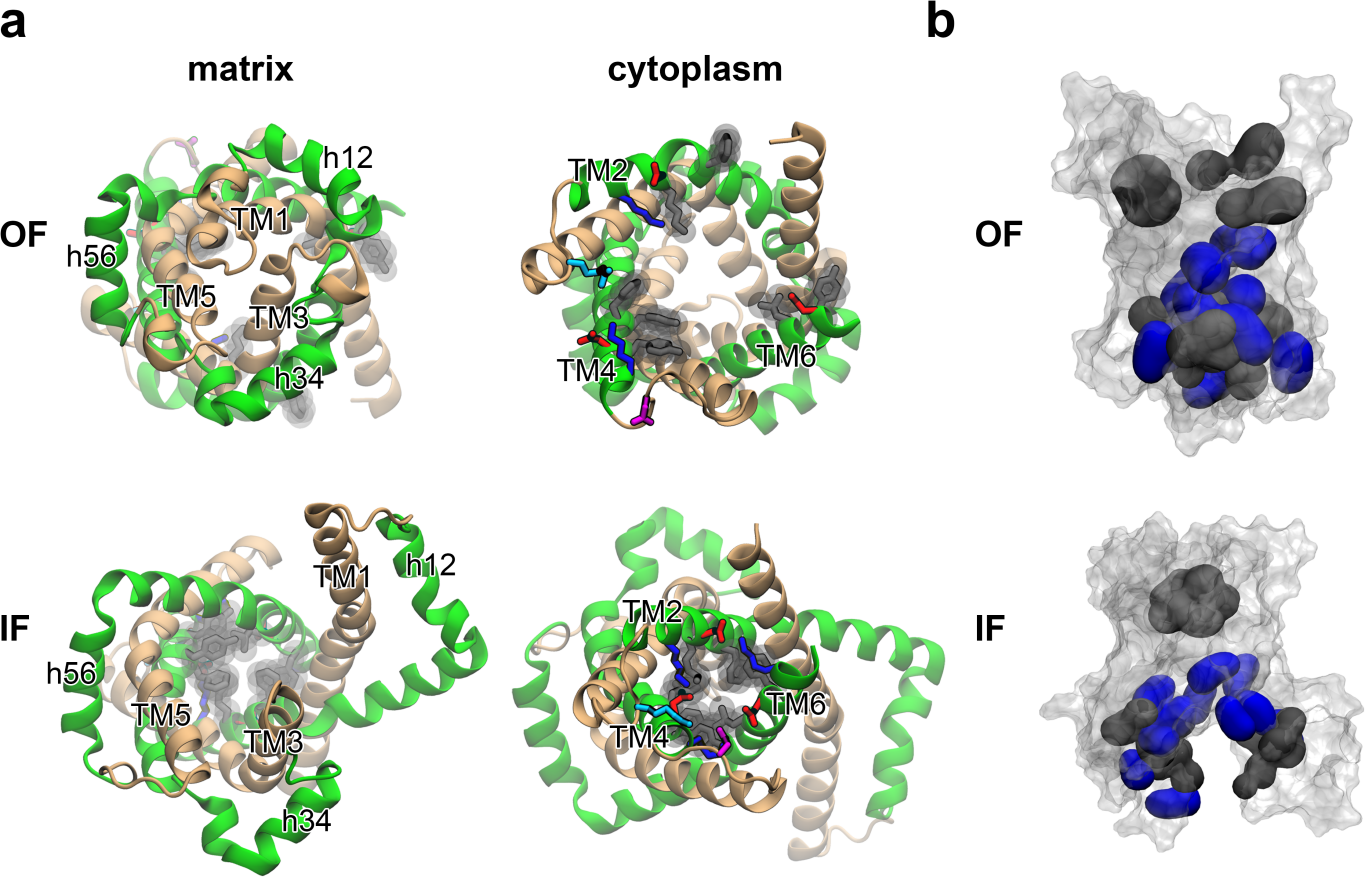
Alternating access mechanism of AAC. (*a*) X-ray structures in the OF form in views from the matrix side (left) and the cytoplasmic one (right) are shown in upper panels, and the last snapshots of APO1 simulation in the IF form are shown in lower panels. Odd-numbered helices are colored in wheat and even-numbered ones in green, respectively. Residues comprising the cytoplasmic salt-bridge network in the IF form are drawn in stick representation in red for acidic residues and in blue for basic ones. Asp203 and Arg104 are colored in magenta and cyan, respectively. Residues constituting the cytoplasmic hydrophobic packing in the IF form are drawn in gray stick and vdW sphere representations. (*b*) Hydrophobic residues comprising the matrix hydrophobic core in the OF form and the cytoplasmic one in the IF form are shown in black surface representation. Basic residues participating in the binding of ADP or BA are shown in blue surface representation.

The predicted IF structure exhibits characteristic features in terms of structural symmetry. In the cytoplasmic side, the even-numbered helices came closer three-fold symmetrically through their large orientational movements, and closed the cytoplasmic channel by the newly formed extensive inter-domain hydrophobic packing and salt-bridge network constituted by the sequentially well-conserved residues [16,29,31] as mentioned above (Fig 8). The observed symmetry of the cytoplasmic side is consistent with the symmetry analysis in a previous study [16]. However, the symmetric closure of the cytoplasmic channel was fulfilled by the formation of tight interactions among the even-numbered helixes (Fig 8), which did not involve apparent twisting motion assumed in the rotary twist model [16].

On the other hand, the orientational movements of the even-numbered helices along with the introductions of kinks at highly conserved kinkable sites pulled outward the odd-numbered ones in the matrix side which closed the matrix channel in the OF form, leading to the wide opening of the matrix pore (Fig 8*A*) consistent with experimental evidences of hydrogen/deuterium exchange and mass spectroscopy [43]. The widely opened matrix domains in the IF form featured large flexibility as represented by large fluctuation of a radius of gyration of the matrix channel [42] (Fig K in S2 Text), while the cytoplasmic half of the IF structure was rigidly maintained as described above. The backward transition from the IF form to the OF one is considered to be a straightforward reverse of the forward process because no disintegration of the secondary structures other than introduction of the local kinks was observed in the forward transition and the large flexibility of the matrix domains allows the matrix channel to be efficiently closed as seen for the binding of the BA inhibitor. The high flexibility of the matrix half of the IF conformation is also suggested to hinder tight packing of the proteins in a crystal phase and may explain the difficulty in determination of a crystallographic structure of the IF form.

In contrast to the nearly symmetric cytoplasmic packing in the IF form, the conformational transition from the OF form to the IF one was observed to be prominently asymmetric; the large orientational movement of domain-I took place in the opening of the matrix pore despite that the perturbative forces were applied symmetrically. The isolation of domain-I in the matrix salt-bridge network upon the binding of ADP observed in the present simulation (Fig 1*E*) may contribute to the asymmetry of the orientational movements. The asymmetric movement appearing at domain-I is consistent with the experimental evidence of a NMR relaxation-dispersion measurement [17] showing that domain-I is more mobile than the others upon binding of ADP substrate and CATR inhibitor, although a clear atomistic correspondence between the observations of the present simulation and the NMR measurement is still missing. A definitive elucidation of the transition path of the alternating access (see below) would be necessary for the purpose.

The orientational movement of domain-I was accompanied by the characteristic dissociation of the inter-domain salt-bridge between Glu29 and Arg279. In the dissociation process, the adenine ring of ADP, of which position and orientation are fixed due to strong salt-bridges of the phosphate moiety of ADP with the matrix basic residues and confinement in the newly created cavity, participated to form a hydrogen-bond between its amino group and Glu29 (Fig 4*E*). Since the salt-bridge is present and absent in the OF form and the IF one, respectively (Fig 4*B* and Figs B*d* and C in S2 Text), the dissociation event can be the crossing of the transition state of the conformational interconversion of the alternating access. The adenine ring is therefore proposed to catalyze the dissociation of the salt-bridge and thus the conformational interconversion.

The proposed catalysis of the adenine ring well explains the strict transport selectivity of the adenosine nucleotides over guanosine and inosine ones [23–25] despite their ability of binding to AAC [24]. MD simulations with a guanosine diphosphate replacing ADP in the transition period of 2,000–2,100 ns in LRPF2 simulation showed that significant reduction of the interaction of the guanine ring with Glu29 (Fig L in S2 Text). The guanine ring was unable to form a stable interaction with Glu29 because of a carboxyl group at the position 6 in the guanine ring, and in the hypoxanthine ring of inosine as well, while the amino group at the position 6 in the adenine ring maintained a stable hydrogen-bond with Glu29 during the transition period as described above (Fig L in S2 Text). Moreover, the amino group at the position 2 in the guanine ring, which is absent in the hypoxanthine ring, did not have a clear partner of interaction with the protein. Since both of the inward and outward transport processes can share the same transition state, the catalysis of the adenine ring also well explains the bidirectionality of the transport selectivity.

The predicted IF form was shown to bind ATP and BA in the matrix pore. The hydrophobic residues located in the pore, which constituted the extensive hydrophobic packing in the matrix side of the OF form and were widely exposed by the opening of the matrix channel upon the formation of the IF form, together with the basic residues in the nucleotide binding site contributed to the binding of BA possessing both the hydrophobic chain and the negatively charged terminal carboxylates (Fig 8*B*). The flexibility of the matrix domains in the IF form was also observed to play a role in capturing BA through the hydrophobic contacts. It is noteworthy that, apart from the identification of the final binding pose of ATP and BA to the IF form which could, in general, require a computational time inaccessible with currently available computational resource, the IF model is the firstly proposed atomic structure which was actually shown to bind ATP and BA from the matrix side.

Recently, an atomic model of an IF form nearly keeping the three-fold symmetry was proposed by meta-dynamics MD simulations [42]. As mentioned above, however, the radius of gyration at the cytoplasmic gate, which is supposed to be closed in the IF form of alternative access, remained considerably large (12 Å) compared to that of the present model (9 Å). Based on the present simulation, therefore, it is likely that the cytoplasmic gate of the previously proposed model [42] is still open and allows formation of a water chain throughout the entire membrane channel (see Fig 3 and Fig K in S2 Text), which may be inconsistent with the fact that proton leakage needs to be avoided at the mitochondrial inner membrane. Furthermore, lack of packing closing the cytoplasmic gate may not sufficiently stabilize the IF conformation as shown in the long-time MD simulation starting from a conformation without the tight packing at the cytoplasmic gate in the present study (see Fig D in S2 Text). In general, any biasing MD simulations including meta-dynamics and LRPF simulations could introduce artefactual conformational changes due to high dimensionality of conformational space. Careful assessment of the modeled structure by long-time MD simulations without biasing is therefore necessary. The present IF model was clearly shown to maintain the highly stable conformation during the long-time unbiased MD simulation for 4 μs because of the well-organized and highly packed hydrophobic and salt-bridge interactions, and also to be capable of binding ATP and BA.

## Conclusions

The present MD simulations with the LRPF method have succeeded in atomistically modeling an IF structure of AAC, which has not been determined experimentally, from the known OF structure through MD simulations of unprecedentedly large conformational changes without a prior knowledge of the target structure. The modeled structure was characterized by well-organized and tight hydrophobic packing and salt-bridges in the cytoplasmic half, and was shown to be very stable in long-time MD simulations. The modeled structure was also shown to spontaneously bind ATP substrate molecule as well as an inhibiter, BA, selectively bound to the IF state as seen experimentally. The present study provides an opportunity for rational design of inhibitors that bind to the IF form of which the atomic structure is experimentally unknown.

Since the LRPF method is designed to provide an approximate path of very large conformational changes without a target structure rather than to precisely determine the minimum free energy path of limited conformational changes, the conformational transition path of the alternating access found in the present simulation were not perfectly resolved. For example, a clear occlude state expected in alternating access transition was missing in the present simulation. Due to the approximate nature of the transition path, it is not possible to determine whether the alternating access involves an occlude state or it proceeds in a manner of direct two-state transition without an occlude state. Nevertheless, the high conformational stability of the modeled IF structure and the proper property of the spontaneous binding of ATP substrate to it enable us to perform LRPF MD simulation of the inverse process from the IF structure to the OF one as well as a path sampling simulation of the alternating access transition along with calculation of free energy profile, which will provide a definitive insight into the conformational changes of alternating access. Although such simulations demand formidable computational resources because of the unprecedentedly large conformational changes of the alternating access transition revealed, some of those simulations are ongoing in the author’s group.

## Materials and methods

We briefly describe the simulation systems and protocols. Detailed explanations are found in Supporting Information (S1 Text).

All energy minimizations and trajectory calculations were done with NAMD [44], except for one of LRPF simulations, LRPF4, which was done with MARBLE [45]. The full-length outward-facing structural model was built based on the X-ray structure of AAC (PDB entry 1OKC) [12]. An ADP molecule was located outside the entrance of the protein. The hydrated bilayer/protein system was subjected to energy minimizations and equilibrations for ~500 ns (Fig 1).

### LRPF simulations

LRPF simulations were performed as described in Ref. 20. A LRPF simulation is constituted by a series of cycles of MD simulation. In each cycle, a biased MD simulation employing biasing forces (Fig 2) is followed by an unbiased MD simulation. A variance-covariance matrix used to calculate the biasing forces from the perturbative forces based on the linear response theory [22] is obtained from the unbiased simulation. The first and the second perturbative forces acting to C_α_ atoms of the acidic amino acids in the MCF motifs (Fig 2*A*) and the charged groups in the cytoplasmic motifs (Fig 2*B*), respectively, were obtained from the average structure calculated from the unbiased trajectory.

### Binding of BA to Apo IF AAC

The force field parameters for bongkrekic acid were generated with paramchem web server (www.paramchem.org). The initial structure of BA was retrieved from ZINC server (zinc.docking.org, ID 4994997). We assumed that BA was fully deprotonated. A snapshot at 2,989 ns in APO3 simulation was used for the binding simulations in which a BA molecule was embedded. We considered a total of 24 configurations of BA (BA1–24); two orientation of BA relative to the protein in which MCT or DCT head group points toward the protein, four rotamer angles around the z axis (0°, 90°, 180° and 270°), and three different distances from the membrane space (10 Å, 15 Å and 20 Å).

### Binding of BA to Apo OF AAC

We also performed simulations of binding of BA to the OF form for comparison, although the binding was not observed experimentally. We removed ADP from the membrane-embedded full-length OF AAC system described above to prepare a membrane-embedded apo OF AAC system. The system was subjected to energy minimizations and equilibrations for ~400 ns. The last snapshot of the trajectory was used for the binding simulations. A BA molecule was aligned to the z axis and located at the entrance of the pore. We considered a total of 8 configurations of BA (BA25–32); two orientation of BA relative to the protein, four rotamer angles around the z axis and a distance from membrane space (10 Å).

## Supporting information

S1 Text Detailed explanation for the simulation setup and LRPF simulations. (PDF)

S2 Text Supplementary simulation results. (PDF)

S1 Movie Conformational transition from the ADP-bound OF form to the ADP-bound IF one. A series of MD simulations of LRPF2, LRPF2+ and MD1 are shown in a concatenated trajectory. ADP is not shown.
(MP4)

S2 Movie An unbiased MD simulation of the apo IF form. The trajectory of APO1 simulation is shown.
(MPG)

S3 Movie Binding of BA to the matrix pore of the IF form in BA8 simulation. BA is drawn in stick representation. Hydrophobic residues in contact with BA are shown in black vdW sphere representation.
(MPG)

S4 Movie Binding of BA to the matrix pore of the IF form in BA16 simulation. BA is drawn in stick representation. Hydrophobic residues in contact with BA are shown in black vdW sphere representation.
(MPG)

S5 Movie Binding of BA to the matrix pore of the IF form in BA20 simulation. BA is drawn in stick representation. Hydrophobic residues in contact with BA are shown in black vdW sphere representation.
(MPG)
