# Supplementary material for: Atomistic modeling of alternating access of a mitochondrial ADP/ATP membrane transporter with molecular simulations
Source: PLoS One. 2017 Jul 20;12(7):e0181489. doi: 10.1371/journal.pone.0181489 (PMC5519185; doi:10.1371/journal.pone.0181489)
Supplement: S1 Text — (PDF) [file pone.0181489.s001.pdf]

Supporting Information

Atomistic Modeling of Alternating Access of a Mitochondrial ADP/ATP Membrane Transporter with Molecular Simulations

Koichi Tamura and Shigehiko Hayashi*

Department of Chemistry, Graduate School of Science, Kyoto University, Kyoto 606-8502, Japan

Email: hayashig@kuchem.kyoto-u.ac.jp

Phone: +81-75-753-4006. Fax: +81-75-753-4000

S1 Text

Supporting Methods

All energy minimizations and trajectory calculations were done with NAMD [44], except for LRPF4 simulation, which was done with MARBLE [45]. The conjugate gradient method was employed for the energy minimizations. The CHARMM22/27 force field [46, 47] with the CMAP correction [48] was adopted for the protein and ADP. Force field parameters for water molecules and lipids were TIP3P [46, 49] and CHARMM36 [50], respectively. Short-range non-bonded interactions were cutoff at 8 Å with the force-switching function beginning at 6.0 Å [51]. Long-range electrostatic interactions under periodic boundary conditions were calculated with the particle mesh Ewald method [52], with a maximum grid spacing maintained within 1 Å. The temperature and the pressure were controlled with Langevin dynamics and the Langevin piston method [53, 54], respectively. In regulation of the pressure, either all the axes were independently scaled (NPT) or the x and y axes were isotropically scaled (NPTiso), where the z axis was perpendicular to the membrane surface. The internal degrees of freedom of water molecules were constrained with the SETTLE algorithm [55]. Bonds including a hydrogen atom were constrained with the RATTLE method [56].

In LRPF4 simulation, the switching function was not applied. Furthermore, the NPTiso ensemble was calculated with an extended system approach of Refs. 45 and 53. Bonds and angles including hydrogen atoms were constrained with the symplectic integrator for rigid bodies [45].

All simulations employed the constant NPTiso (298 K, 1 bar) condition unless otherwise noted. Fig 2 were prepared with CCP4mg [57]. Other molecular figures were generated with VMD [58].

Modeling full-length outward-facing structure of AAC. The full-length outward-facing structural model was based on the X-ray structure reported in 2003 (PDB entry 1OKC) [12]. Bound water molecules were retained, whereas CATR and cardiolipins were not included in the model. Missing residues (Ser1, Lys294, Lys295, Phe296 and Val297) were added using PyMOL [59]. Hydrogen atoms were added using the psfgen plugin in NAMD. The titration states of ionizable residues were assumed to be those at pH 7, except for those of histidine residues which were determined based on visual inspection. In the X-ray structure, His39 is surrounded by two acidic residues and therefore it was assumed to be positively charged. Other histidine residues were assumed to be the neutral N_δ tautomer. Then, the isolated protein was energetically minimized with the modeled part only being allowed to move.

Modeling membrane-embedded outward-facing AAC. The modeled full-length AAC was manually embedded in a 100 Å × 100 Å palmitoyloleoylphosphatidylcholine (POPC) bilayer modeled using VMD. An ADP molecule was located above the entrance of the protein. The structure of ADP was taken from PDB entry 2HYD. The bilayer was solvated with TIP3P water molecules using the solvate plugin in VMD. To neutralize the system, 17 chloride ions were randomly added to the bulk solvent using the autoionize plugin in VMD. The simulation box was initially 100 Å × 100 Å × 90 Å in size and contained 78,895 atoms.

Preliminary equilibration of the ADP-added outward-facing AAC. The system was subjected to energy

minimizations and equilibrations as follows. The time step of trajectory calculation was set to 1 fs. First, a 5,000-step energy minimization and a 500-ps MD simulation under the NVT condition were performed with lipid tails only being allowed to move. The same procedure was repeated except that only lipids were allowed to move. Then, a 5,000-step energy minimization, a 300-ps temperature annealing simulation and a 500-ps MD simulation were performed at the NPT condition with the protein backbone and ADP being harmonically restrained with a force constant of 100 kcal/mol/Å^2. Finally, the restraint was removed, followed by a 10,000-step energy minimization, a 300-ps annealing simulation and a 500-ps equilibrium MD simulation at the NPT condition.

Further equilibration of the ADP-added outward-facing AAC. We further performed a 215-ns MD simulation started from the last snapshot of the pre-equilibrated system. The time step of trajectory calculation was set to 2 fs and the constant NPT condition was employed. We did not employ NPTiso condition in the early stage of equilibration as the additional constraint of the pressure control of NPTiso condition could fail in detecting and removing artifact of the protein-membrane structure inevitably introduced in the modeling of the simulation system. In fact, extremely anisotropical deformation (~80 Å × 120 Å) of the membrane surface was observed in the course of the simulation. As the deformation could lead to the unwanted contact of the protein with its own periodic image, we discarded the bilayer and generated a new system as follows. First, the protein and ADP, together with water molecules and chloride ions within 5 Å of the protein, were extracted from the last snapshot of the 215-ns MD trajectory and were manually embedded in a newly generated 100 Å × 100 Å POPC bilayer. The bilayer was solvated and neutralized to generate a simulation box with an initial size of 100 Å × 100 Å × 90 Å. The box contained 80,600 atoms.

The system was subjected to energy minimizations and equilibrations as follows. The time step was set to 1 fs. First, a 5,000-step energy minimization and a 1-ns MD simulation under the NVT condition were performed with lipid tails only being allowed to move. The same procedure was repeated except that only lipids were allowed to move. Then, a 5,000-step energy minimization, a 300-ps annealing simulation and a 20-ns MD simulation at the NPT condition was performed, with the heavy atoms of the protein and ADP being harmonically restrained with a force constant of 100 kcal/mol/Å^2. Hereafter, the time step of 2 fs was adopted. A 5-ns MD simulation where the restraint on the side chain was gradually removed was performed, followed by a 20-ns MD simulation at the NPT condition in which the restraint on the protein backbone and ADP was still retained. The force constant of the restraint was gradually reduced to 1 kcal/mol/Å^2 in the course of the subsequent 3.5-ns MD simulation, followed by a 20-ns MD simulation at the NPT condition in which the restraint was retained. Finally, the restraint was gradually decreased to zero in the course of the subsequent 2-ns MD simulation at the NPT condition. Then, a 300-ns MD simulation without any restraint was performed at the NPTiso condition. The resultant trajectory was combined with the 215-ns MD trajectory mentioned above and regarded as an equilibrium trajectory wherein ADP bound to the protein (Fig 1).

LRPF simulations. LRPF simulations were performed as described in Ref. 20. In LRPF simulations, directed biasing forces are applied to protein atoms to induce functionally relevant large and global conformational changes of the protein during MD simulations. The directed biasing forces were determined based on the linear response theory [22] that defines

protein's global conformational response to local perturbative forces related to its function such as ligand binding and enzymatic reaction. Despite the local nature of the perturbative forces, the protein response is often observed to appear extensively in the protein structure [20, 22]. The local-global coupling is thus exploited to define a reaction coordinate of the global conformational changes of the functional process, which is usually difficult to define because of its global nature. Non-linearity of the conformational changes is then taken into account by an iterative procedure that updates the linear response biasing forces during the MD simulations [20]. The LRPF method allows one to simulate and predict the large and global conformational changes of the functional process without a priori knowledge of the target protein structure, since the biasing forces that induce the global conformational changes are determined based on the functionally related local perturbative forces that can often be deduced from biological and biochemical considerations.

A LRPF simulation is constituted by a series of cycles of MD simulations. In each cycle, a biased MD simulation is followed by an unbiased MD simulation. The biased MD simulation consists of three consecutive phases. In the first phase, the biasing forces are gradually grown in the course of the simulation. When the magnitude of biasing forces reaches a sufficient level, the first phase is ended and the second phase where the constant biasing forces are applied is started. The second phase is ended when RMSD reaches plateau around 1.5−2 Å (see below), followed by the third phase where the biasing forces are gradually decreased. The unbiased simulation is started when the biasing forces are turned off. The unbiased simulation is lasted until RMSD reaches plateau, and then the next cycle is started. A variance-covariance matrix needed to calculate the biasing forces from the perturbative forces based on the linear response theory is obtained from the unbiased simulation.

In the LRPF simulation, one needs to determine the following parameters that control the protocol, i.e., the expected RMSD induced in a cycle of a biasing phase, the magnitude of the biasing force, and the simulation times of the biasing phase and the unbiasing one. In principle, the smaller expected RMSD, the weaker biasing force, and the longer simulation times add less perturbation by the biasing force and thus give a more accurate trajectory closer to the minimum free energy path. However, such simulation obviously requires a larger computational resource. Furthermore, the appropriate values of those parameters can vary depending on the protein treated. One therefore needs to choose well-balanced values of the parameters for the target protein through adjustment by preliminary calculations.

The expected RMSD is set to be sufficiently large to efficiently induce conformational changes but to be sufficiently small to be in a linear response regime. In our experience, the expected RMSD of 1.5–2 Å seems to be a reasonable choice. Then, based on a metric of work (W), which is the product of the expected RMSD by the biasing force, the magnitude of the biasing force is preliminary determined by the work which is roughly comparable to the expected activation energy for the conformational changes (typically 10–20 kcal/mol). The biasing force is then reduced so as to keep the RMSD around 1.5–2 Å at plateau in the biasing simulation. The simulation time for the relaxation phase is also determined by inspection of temporal behavior of RMSD. More detailed discussions on those control parameters are found in a previous report [20].

Three snapshots were taken from the 300-ns equilibrium trajectory from which LRPF simulations were started. The first cycles of LRPF1, LRPF2 and LRPF3 were started from the snapshot at 280 ns, 290 ns and 300 ns, respectively. A 270–280 ns, 280−290 ns and 290−300 ns portions of the 300-ns trajectory were used to calculate the initial variance-

covariance matrices in LRPF1, LRPR2 and LRPF3, respectively. LRPF4 computed with MARBLE was also started from the snapshot at 300 ns. LRPF1, LRPF2, LRPF3, LRPF4 and LRPF2+ simulation took 22, 17, 43, 27, and 2 cycles, respectively. Typically, a biased and an unbiased simulation of a LRPF cycle took 40 and 50 ns, respectively.

The first perturbative forces acting to C_α atoms of the acidic amino acids in the MCF motifs, i.e., Glu29, Asp134, and Asp231 (Fig 2*a*), were obtained as follows. First, the last 10-ns portion of the unbiased trajectory in a LRPF cycle was used to calculate an average structure and a variance-covariance matrix. Then, using the structure, we calculated the center of the geometry (COG) of the pyrophosphate moiety and three vectors, $\boldsymbol{A}$, $\boldsymbol{B}$ and $\boldsymbol{C}$, which point from the COG toward C_α atom of Glu29, Asp134 and Asp231, respectively. The magnitude of each vector was set to be inversely proportional to the square of the distance between the COG and C_α atom. These vectors were employed as the perturbative forces and converted to the biasing forces (Fig 2*a*) by taking the product with the covariance matrix. The biasing forces were scaled as described in Ref 20.

The second perturbative forces acting to C_α atoms of the charged groups in the cytoplasmic motifs (Fig 2*b*) were calculated as follows. First, we calculated an average structure and a covariance matrix from the last 10-ns portion of the unbiased trajectory of a LRPF cycle. Then, for each pair of residues of the cytoplasmic network, vectors $\overrightarrow{AB}$ and $\overrightarrow{BA}$ were calculated from the average structure where A and B represent the positions of the paired residues. These vectors were regarded as the second perturbative forces. The biasing forces obtained with the perturbative forces were also scaled as described in Ref 20.

SMD simulations. ADP was forced to dissociate from the protein by steered MD (SMD) simulations [60]. In the SMD simulations, the center of mass (COM) of the pyrophosphate moiety was pulled toward the matrix side by applying a harmonic restraint moving along the z axis. The rate of the moving restraint was set to be 0.075 Å/ns and the force constant was 2 kcal/mol/Å^2. On the other hand, in the SMD simulations which subsequently led to APO3 and APO4 simulations, the adenine moiety was pulled down as the adenine moiety was exposed to the solvent. In all the SMD simulations, C_α atoms of chosen cytoplasmic residues were harmonically restraint along the z axis with a force constant being 10 kcal/mol/Å^2. The residue numbers were 5, 97, 108, 202, 209 and 292 [26].

Binding of ATP to apo IF AAC. The initial structure of ATP was adopted from PDB entry 4Q4A. The force field parameters of ATP were taken from Komuro, et al. [61]. The last snapshot of APO1 simulation was used for the binding simulations. Three chloride ions bound inside the protein and one in the bulk solvent were removed and ATP was put onto the entrance of the matrix cavity. We considered 8 configurations of ATP (ATP1-8); two orientation of ATP relative to the protein in which triphosphate or adenine moiety points toward the protein, four rotamer angles around the z axis (0˚, 90˚, 180˚ and 270˚), and a distance from membrane space (10 Å).

Binding of BA to apo IF AAC. The force field parameters for bongkrekic acid were generated with paramchem web server (www.paramchem.org). The initial structure of BA was retrieved from ZINC server (zinc.docking.org, ID 4994997). We assumed that BA was fully deprotonated. The isolated BA was immersed in a rectangular box of size 50

Å × 50 Å × 50 Å filled with TIP3P water molecules. Three sodium ions were randomly added to neutralize the system. After the energy minimization, the temperature was gradually raised to 298 K, followed by a 1-ns MD simulation at the NPT condition. Thereafter, a 100-ns MD simulation under the NVT condition was performed. We then calculated the distance between C19 and C24 atoms along the 100-ns trajectory to generate its distribution (atom names correspond to ones in the ZINC file). The distance describes approximate length between MCT and DCT head groups (see Fig 7*a*) and was broadly peaked at ~13 Å (data not shown). The BA structure of the last snapshot of the 100-ns trajectory possesses the distance of 18.0 Å and thus represented an extended form of BA. The axis defined by C19 and C24 atoms of the extended structure was then aligned to the z axis and located at the entrance of the matrix pore. A snapshot at 2,989 ns in APO3 simulation was used for the binding simulations. In this particular snapshot, two chloride ions occupied deeply inside the pore together with several ones at the entrance of the pore. The occupations of the chloride ions in the snapshot was confirmed to represent the average binding pose of the ions; in APO1 trajectory, two chloride ions occupied in average (data not shown). Water molecules overlapping with the BA were removed. We considered a total of 24 configurations of BA (BA1−24); two orientation of BA relative to the protein in which MCT or DCT head group points toward the protein, four rotamer angles around the z axis (0˚, 90˚, 180˚ and 270˚), and three different distances from the membrane space (10 Å, 15 Å and 20 Å). The distance between the membrane space and BA was determined based on the average z coordinate of phosphorous atoms of lipids at the matrix side and the nearest BA atom. The BA atom was C19 atom when MCT pointed toward the protein or C24 atom otherwise.

Binding of BA to apo OF AAC. We removed ADP from the membrane-embedded full-length OF AAC system described above to prepare a membrane-embedded apo OF AAC system. We adopted the careful procedure for minimizations and equilibrations described above. A 10-ns MD simulation under the NPT condition was started from the equilibrated snapshot. We then carried out a 400-ns MD simulation under the NPTiso condition. The last snapshot of the trajectory was used for the binding simulations. In this particular snapshot, three chloride ions occupied deeply inside the cytoplasmic pore. The structure represented the average binding pose of the ion (data not shown). The last snapshot of the equilibrated BA was aligned to the z axis and located at the entrance of the pore as described above. Water molecules overlapping with the BA were removed. We considered a total of 8 configurations of BA (BA25−32); two orientation of BA relative to the protein, four rotamer angles around the z axis and a distance from membrane space (10 Å). We only considered the distance of 10 Å because the matrix side of the apo OF structure possesses an extensive structural fold and interfered with the periodic image of BA. The structural fold in the IF form were widely opened and the problem was not present for the IF form.

Unbiased simulations of GDP-bound form of AAC. Five snapshots (GDP1−5) were taken from LRPF2 trajectory (snapshot at 2000 + 20 × i ns, i = 0, 1, 2 ,3 ,4) and each ADP in the snapshot was replaced with GDP. 10-ns unbiased simulation was then performed from which nearest-distance distribution in Figure 12 in **S2 Text** was extracted.

Calculation of contact index. Contact index is defined as follows. If distance between residues is less than a certain

threshold value d, then contact equals 1 and zero otherwise. The threshold value is varied according to the residue type involved. When distance between lysine and aspartic/glutamic residues is concerned, then d = 4.5 Å. The distance was measured between N_ζ atom of lysine and C_γ (C_δ) atom of aspartic (glutamic) acid residue. On the other hand, d = 5.0 Å if arginine and the acidic residues are involved. The distance was measured between C_ζ atom of arginine and C_γ (C_δ) atom of aspartic (glutamic) acid residue. Furthermore, the contact index among phenylalanine, tyrosine and leucine was 1 if distance between the COMs of the side chains is less than 5.0 Å and zero otherwise. Contact index between phenylalanine and lysine was 1 if distance between the COMs of the side chains is less than 5.5 Å and zero otherwise. The side chain of lysine did not include the amino group. Contact index between Glu29 and the amino group of ADP was 1 if distance between C_δ atom of Glu29 and the nitrogen atom of the amino group is less than 4.5 Å and zero otherwise.

Calculation of helix angle. We followed Wang and Tajkhorshid to calculate the helix angle [30]. The helix angle of TM1 is Q38−P27'−Q38', one of TM3 is A142−P132'–A142' and one of TM5 is Q240−P229'−Q240'. The prime indicates that the position is that of the X-ray structure. Each snapshot of a trajectory is superimposed to the X-ray structure before the angle calculation.

Calculation of number of water molecules at the matrix side. We calculated the number of water molecules at the matrix side of the protein as follows. In each snapshot of a trajectory we calculated the COM of the protein. Then, the number of water molecules at the matrix side was counted for the water molecules which were within 20 Å of the COM of the protein and whose z coordinate was less than one of the COM. Note that in this definition, bulk water molecules near the matrix side of the protein were always included and therefore the number of water molecules at the matrix side did not vanishes even in the OF state where the matrix side of the protein was tightly packed (Fig 1*c*).

Calculation of radii of gyration of the cytoplasmic and matrix channels. The same definition of the radii of gyration of the cytoplasmic and matrix channels as that introduced in a recent report by Pietropaolo *et al.* [17] was used. The residues for which the radii of gyration were calculated are F11, F88, D92, K95, L116, F191, D195, K198, W213, L287, D291, K294, K91, Y194, and Y290 for the cytoplasmic channel (Fig 9*a* in **S2 Text**), and are P27, E29, K32, G72, P132, D134, R137, G175, P229, D231, R234, and G272 for the matrix one (Fig 9*b* in **S2 Text**).

Supporting References

46. MacKerell AD, Bashford D, Bellott M, Dunbrack RL, Evanseck JD, Field MJ, et al. All-atom empirical potential for molecular modeling and dynamics studies of proteins. J Phys Chem B. 1998; 102: 3586–3616.
47. Foloppe N, MacKerell AD. All-atom empirical force field for nucleic acids: I. Parameter optimization based on small molecule and condensed phase macromolecular target data. J Comput Chem. 2000; 21: 86–104.
48. MacKerell AD Jr, Feig M, Brooks III CL. Extending the treatment of backbone energetics in protein force fields: Limitations of gas-phase quantum mechanics in reproducing protein conformational distributions in molecular dynamics simulations. J Comput Chem. 2004; 25: 1400–1415.
49. Jorgensen WL, Chandrasekhar J, Madura JD, Impey RW, Klein ML. Comparison of simple potential functions for simulating liquid water. J Chem Phys. 1983; 79: 926–935.
50. Klauda JB, Venable RM, Freites JA, O'Connor JW, Tobias DJ, Mondragon-Ramirez C, et al. Update of the CHARMM all-atom additive force field for lipids: Validation on six lipid types. J Phys Chem B. 2010; 114: 7830–7843.
51. Steinbach PJ, Brooks BR. New spherical-cutoff methods for long-range forces in macromolecular simulation. J Comput Chem. 1994; 15: 667–683.
52. Darden T, York D, Pedersen L. Particle mesh Ewald: An N•log(N) method for Ewald sums in large systems. J Chem Phys. 1993; 98: 10089–10092.
53. Martyna GJ, Tobias DJ, Klein ML. Constant pressure molecular dynamics algorithms. J Chem Phys. 1994; 101: 4177–4189.
54. Feller SE, Zhang Y, Pastor RW, Brooks BR. Constant pressure molecular dynamics simulation: The Langevin piston method. J Chem Phys. 1995; 103: 4613–4621.
55. Miyamoto S, Kollman PA. SETTLE: An analytical version of the SHAKE and RATTLE algorithm for rigid water models. J Comput Chem. 1992; 13: 952–962.
56. Andersen HC. Rattle: A "velocity" version of the Shake algorithm for molecular dynamics calculations. J Comput Phys. 1983; 52: 24–34.
57. McNicholas S, Potterton E, Wilson KS, Noble MEM. Presenting your structures: the *CCP4mg* molecular-graphics software. Acta Cryst. 2011; D67: 386–394.
58. Humphrey W, Dalke A, Schulten K. VMD: visual molecular dynamics. J Mol Graphics. 1996; 14: 33–38.
59. The PyMOL Molecular Graphics System, Version 1.2r1 Schrödinger, LLC.
60. Isralewitz B, Gao M, Schulten K. Steered molecular dynamics and mechanical functions of proteins. Curr Opin Struct Biol. 2001; 11: 224–230.
61. Komuro Y, Re S, Kobayashi C, Muneyuki E, Sugita Y. CHARMM force-fields with modified polyphosphate parameters allow stable simulation of the ATP-bound structure of Ca^{2+}-ATPase. J Chem Theory Comput. 2014; 10: 4133–4142.
