# Supplementary material for: Atomistic modeling of alternating access of a mitochondrial ADP/ATP membrane transporter with molecular simulations
Source: PLoS One. 2017 Jul 20;12(7):e0181489. doi: 10.1371/journal.pone.0181489 (PMC5519185; doi:10.1371/journal.pone.0181489)
Supplement: S2 Text — (PDF) [file pone.0181489.s002.pdf]

Supporting Information

Atomistic Modeling of Alternating Access of a Mitochondrial ADP/ATP Membrane Transporter with Molecular Simulations

Koichi Tamura and Shigehiko Hayashi*

Department of Chemistry, Graduate School of Science, Kyoto University, Kyoto 606-8502, Japan

Email: hayashig@kuchem.kyoto-u.ac.jp

Phone: +81-75-753-4006. Fax: +81-75-753-4000

S2 Text

Table 1. Summary of Simulations.

simulation	for what	biasing force	duration (ns)
OFADP	binding of ADP to OF form	no	500
LRPF1	LRPF simulation	$\mathbf{F}^{bias}_{matrix}$	4,897
LRPF2	LRPF simulation	$\mathbf{F}^{bias}_{matrix}$	2,500
LRPF3	LRPF simulation	$\mathbf{F}^{bias}_{matrix}$	4,905
LRPF4	LRPF simulation	$\mathbf{F}^{bias}_{matrix}$	4,052
LRPF2+	LRPF simulation	$\mathbf{F}^{bias}_{cytoplasm}$	154
MD1	equilibration of ADP-bound IF form	no	4,000
SMD	dissociation of ADP from IF form	moving restraining force	500
APO1	equilibration of apo IF form	no	4,000
APO2	equilibration of apo IF form	no	3,000
APO3	equilibration of apo IF form	no	3,000
APO4	equilibration of apo IF form	no	2,000
APO1+	equilibration of apo IF form with elongated cutoff distance	no	2,000
ATP1–8	binding of ATP to IF form	no	160 (= 20 × 8)
BA1–24	binding of BA to IF form	no	480 (= 20 × 24)
BA25–32	binding of BA to OF form	no	160 (= 20 × 8)
BA8	equilibration of BA-bound IF form	no	1,000
BA16	equilibration of BA-bound IF form	no	1,000
BA20	equilibration of BA-bound IF form	no	1,000
GDP	equilibration of GDP-bound form	no	50 (= 10 × 5)
			total 39.358 μs

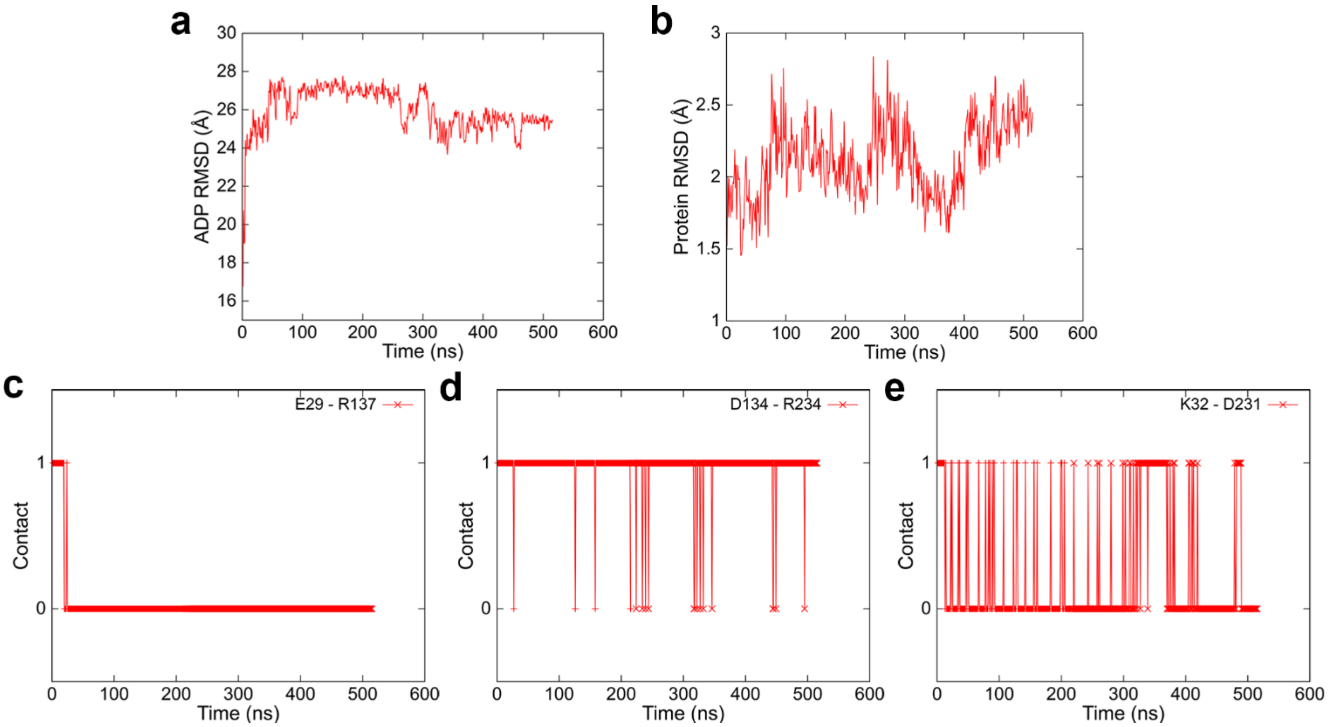

Fig A

Binding of ADP to the OF form. Results of the ADP binding simulation are shown. (*a-e*) Time courses of heavy-atom RMSD of ADP with respect to the initial conformation (*a*), C_{α}-RMSD of the protein with respect to the X-ray structure (PDB entry 1OKC) (*b*), contact between Glu29 and Arg137 (*c*), contact between Asp134 and Arg234 (*d*), and contact between Lys32 and Asp231 (*e*). See **S1 Text** for the definition of the contact between the residues.

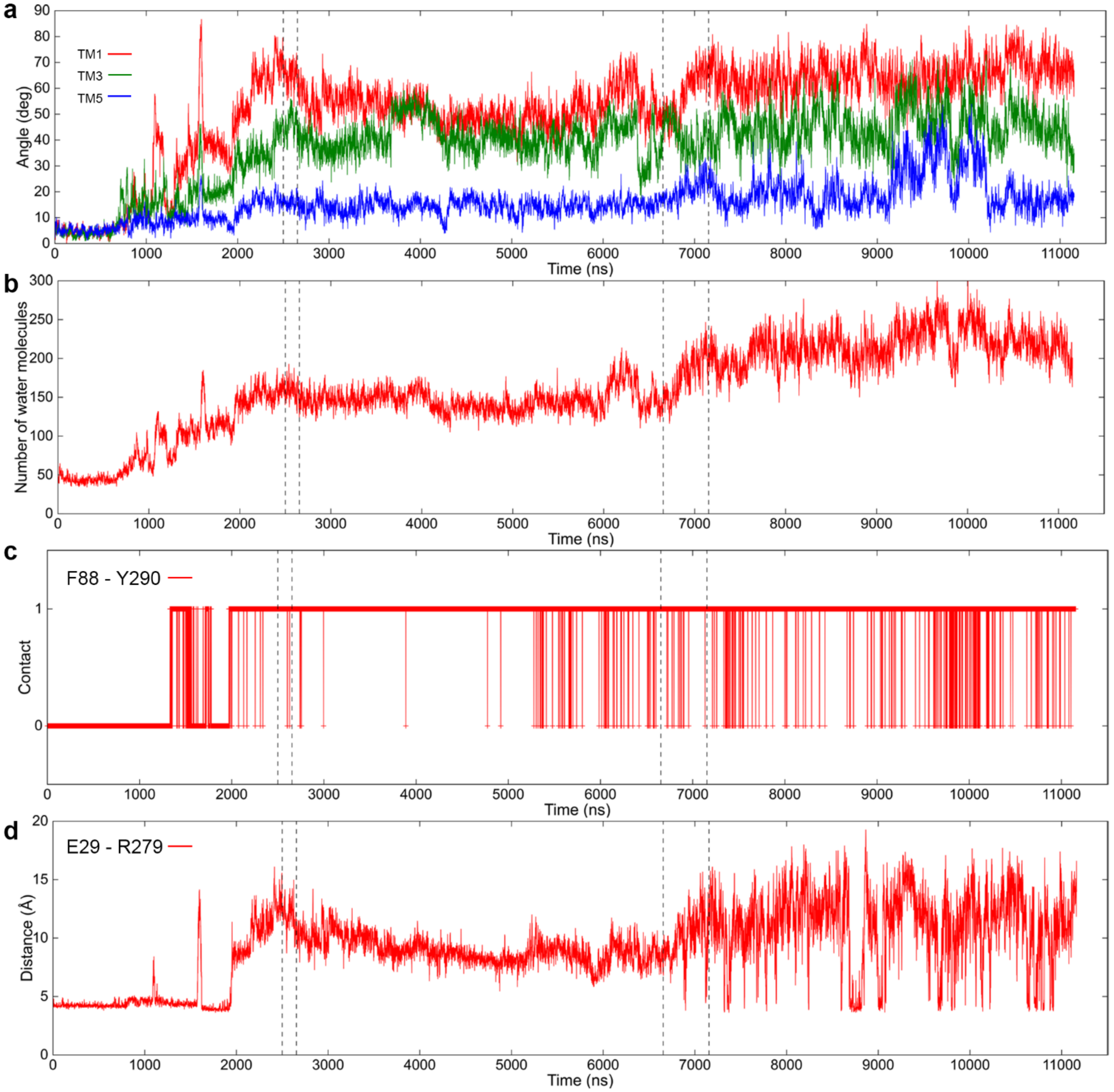

Fig B

Conformational transition from the OF form to the IF one. Results of LRPF2, LRPF2+, MD1, SMD and APO1 simulations are shown. (*a*-*d*) Time courses of helix angles (*a*), the number of water molecules at the matrix side (*b*), contact between Phe88 and Tyr290 (*c*), and distance between Glu29 and Arg279 (*d*). Vertical lines indicate the times at which the simulations were changed (see Fig 3 in the main text). See **S1 Text** for the definition of the parameters.

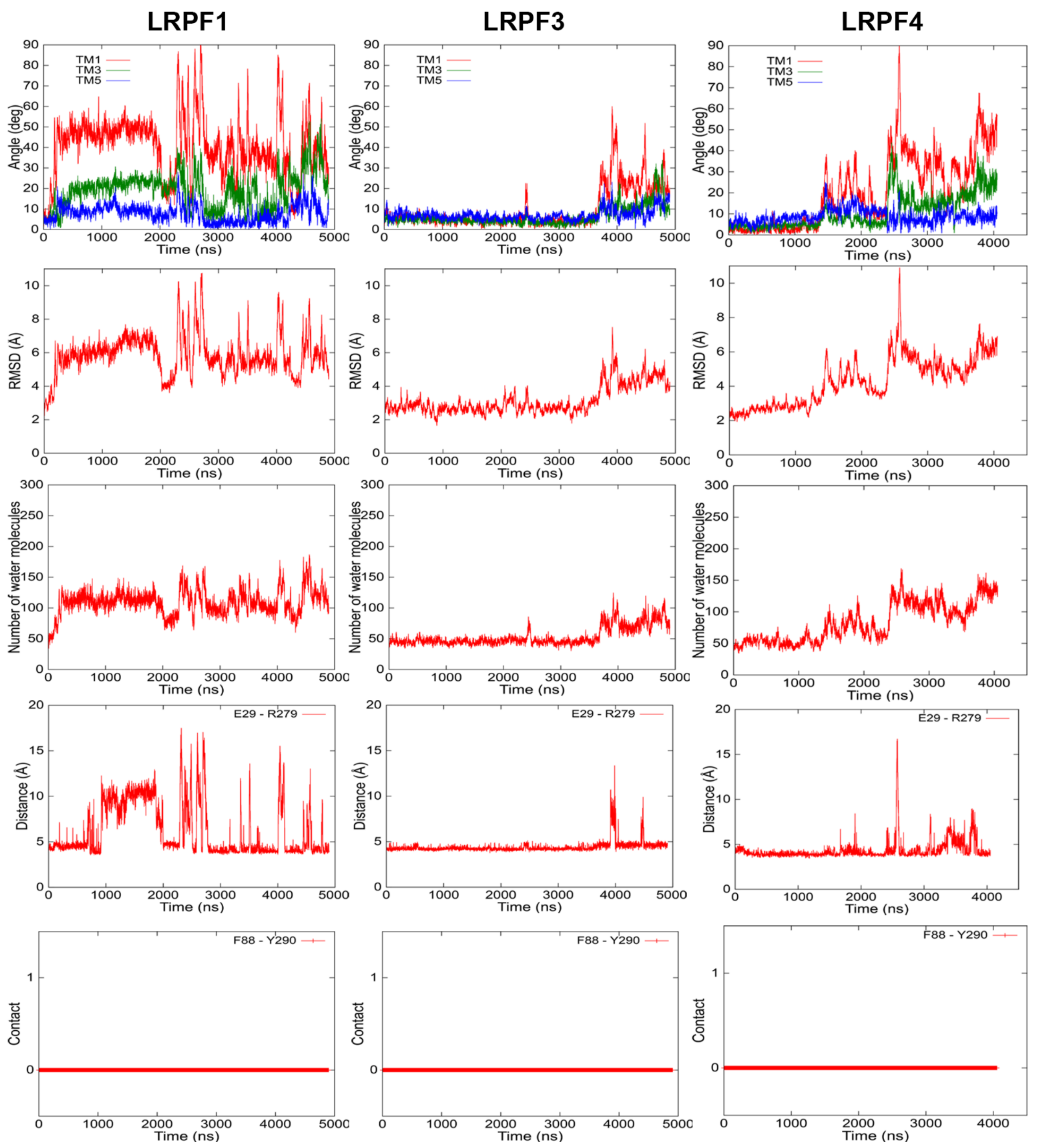

Fig C

Results of LRPF1, LRPF3 and LRPF4 simulations. Time courses of helix angles (top), C_{α}-RMSD of the protein with respect to the X-ray structure (the second row), the number of water molecules at the matrix side (the third row), distance between Glu29 and Arg279 (the fourth row), and contact between Phe88 and Tyr290 (bottom). See **S1 Text** for the definition of the parameters.

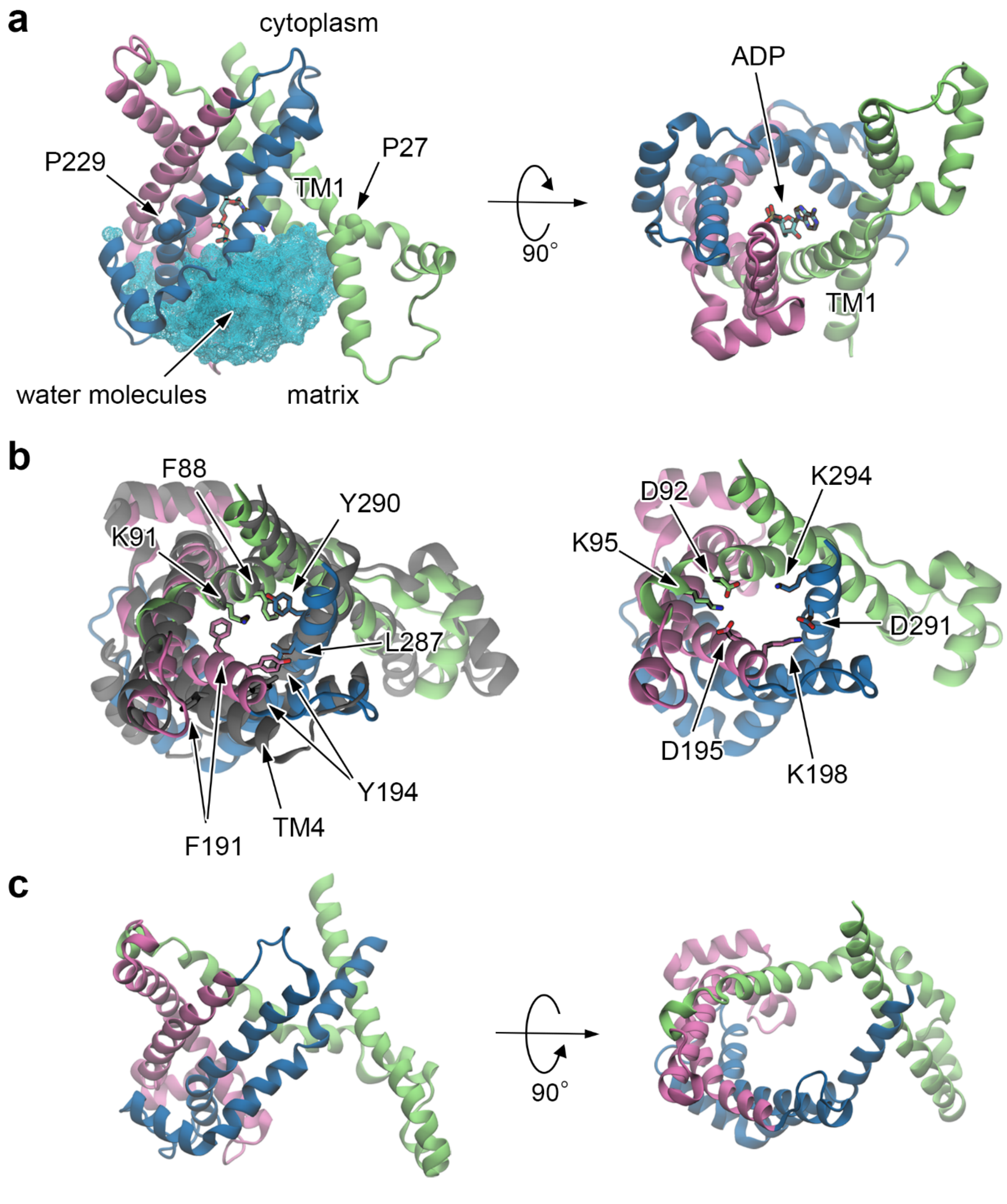

Fig D

Structural changes observed in LRPF2 and LRPF2+ simulations. (*a*) The last snapshot of LRPF2 simulation. Water molecules at the matrix side are drawn in surface representation and colored in cyan. (*b*) Snapshots at the beginning (black) and the end of LRPF2+ simulation, respectively, in a view from the cytoplasmic side are superimposed. (*c*) Collapse of protein structure observed in the last snapshot of the ~3-μs unbiased MD simulation started from the last frame of LRPF2 simulation. ADP is not shown.

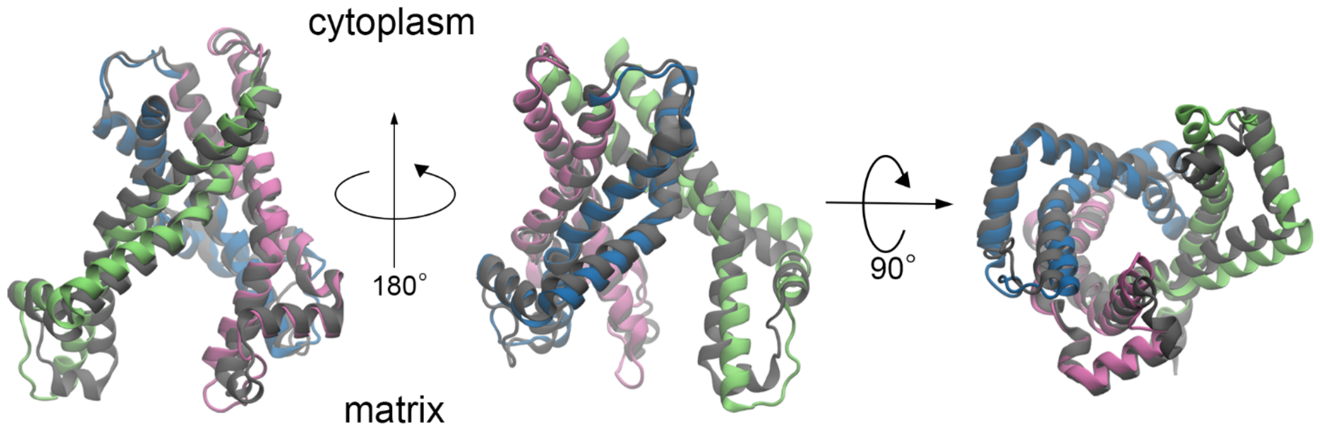

Fig E

The average structures of APO1 and APO2 (black) simulations. APO2 simulation was an unbiased MD simulation for 3 μs starting with an initial structure taken from the structure at 200 ns in the SMD simulation.

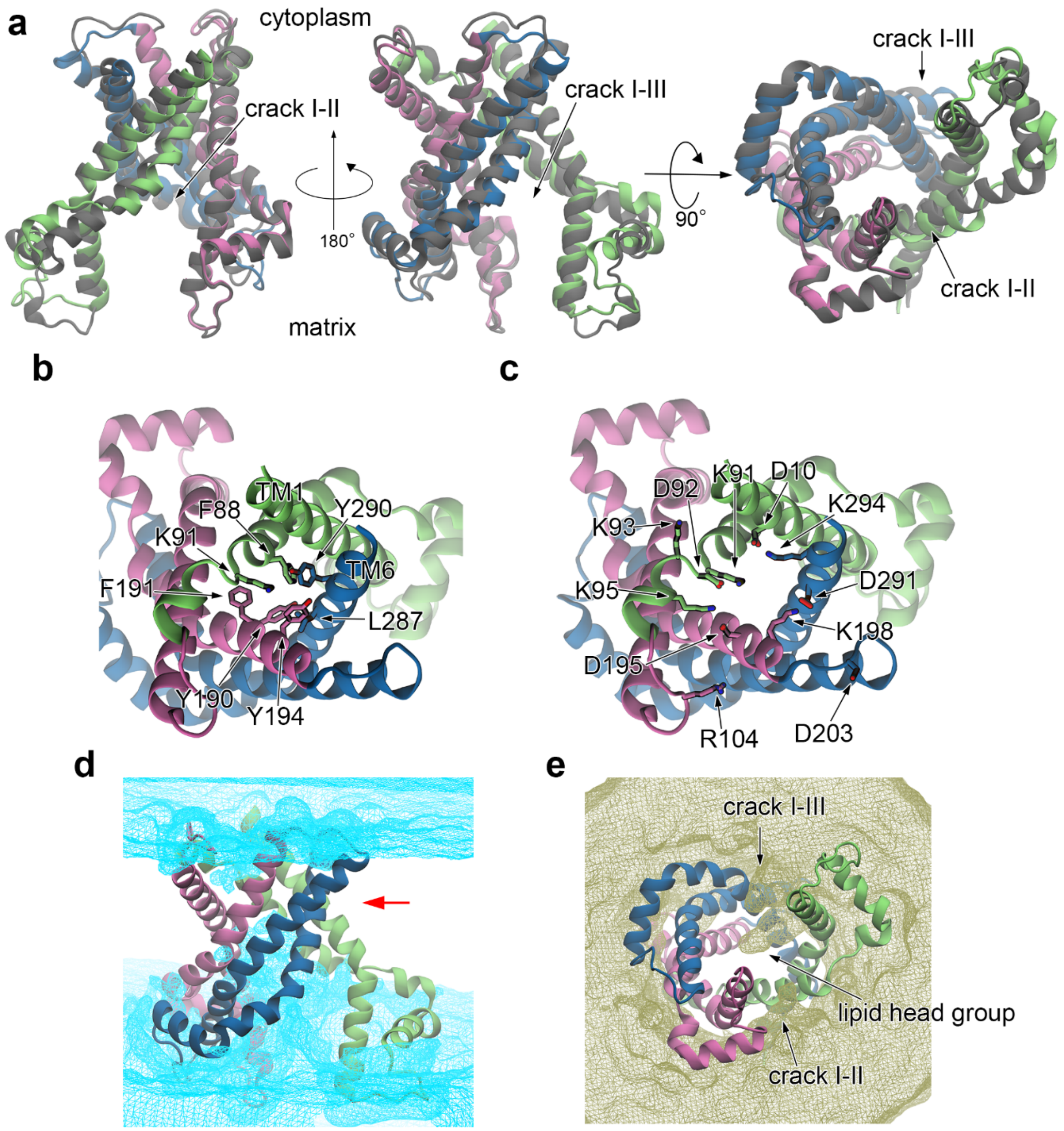

Fig F

Results of APO3 and APO4 simulations. APO3 and APO4 simulations were unbiased MD simulations for 3 μs and 2 μs starting with initial structures taken from the last structures of SMD simulations for ~500 ns starting at 1 μs and 2 μs in MD1 simulation, respectively. In the two structures, therefore, the extensive salt-bridge network in the cytoplasmic side was not completed while the inter-domain hydrophobic packing was already generated. (*a*) The average structures of APO3 and APO4 (black) simulations. (*b,c*) Hydrophobic residues (*b*) and charged ones (*c*) participating in the cytoplasmic inter-domain hydrophobic packing and salt-bridge network in the IF form are shown in views from the cytoplasmic side, respectively. (*d,e*) Distributions of water molecules (*d*) and lipid molecules (*e*) contoured at 0.3 occupancy level calculated from the last 100-ns portion of the trajectory are shown. A red arrow indicates the cytoplasmic constriction site in the IF form.

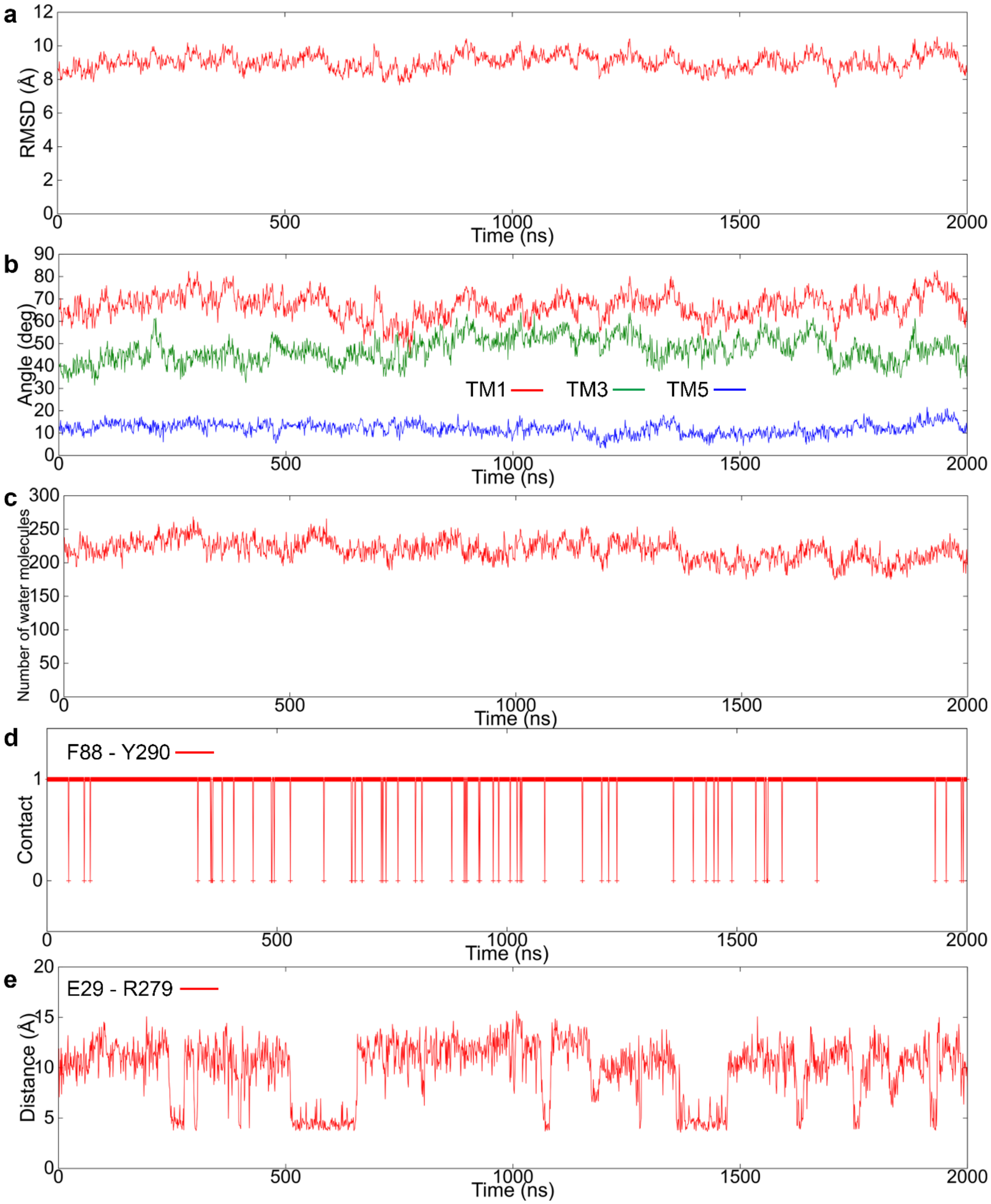

Fig G

MD simulation of the apo IF form with an elongated cutoff distance (APO1+). (*a-e*) Time courses of C_α-RMSD with respect to the X-ray structure (*a*), helix angles (*b*), the number of water molecules at the matrix side (*c*), contact between Phe88 and Tyr290 (*d*), and distance between Glu29 and Arg279 (*e*). See **S1 Text** for the definition of the parameters.

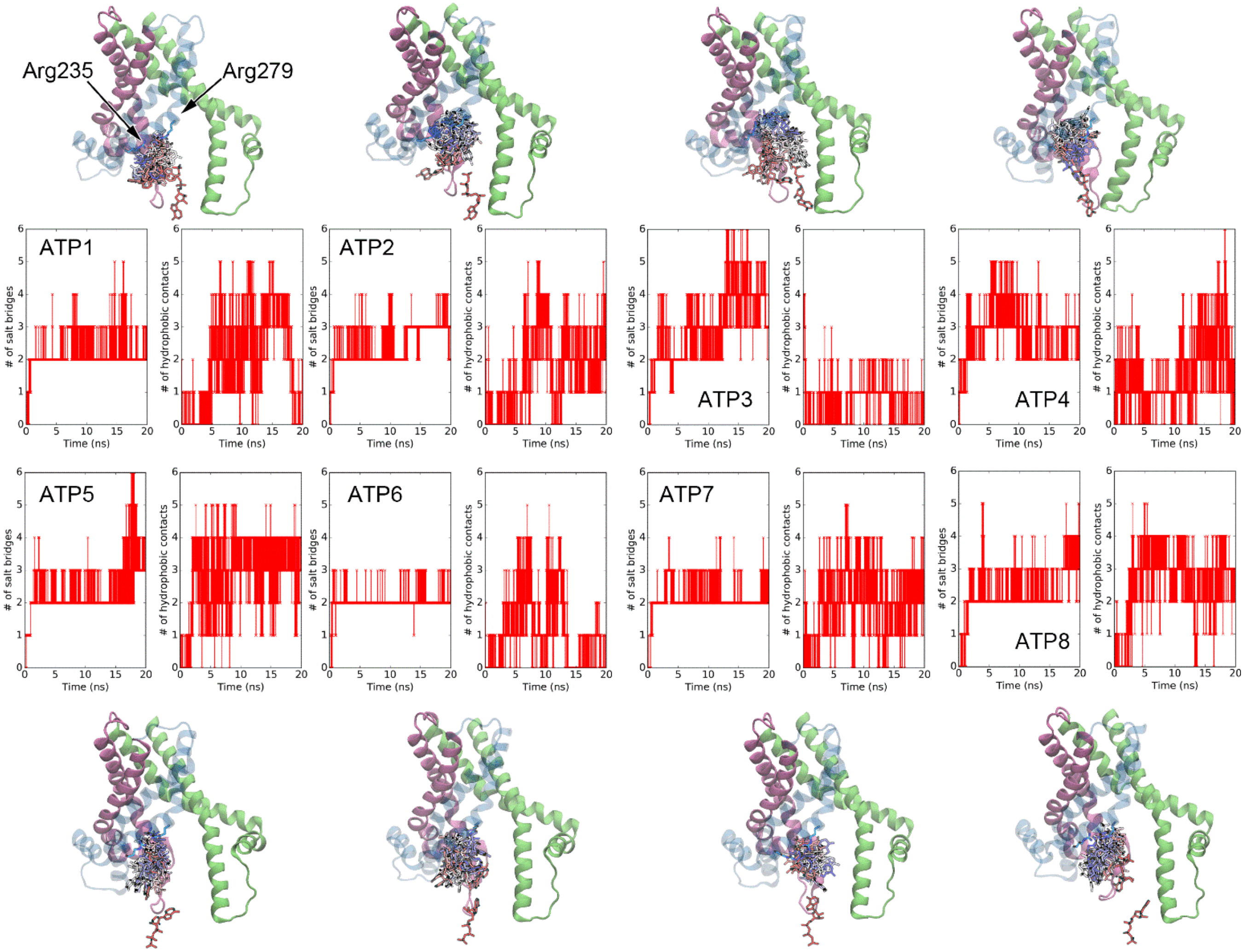

Fig H

Binding simulation of ATP to the IF form (ATP1–8). Time courses of the number of salt-bridges (left) and hydrophobic contacts (right) are shown. Trajectories of ATP at every 1 ns are mapped onto the protein structure of the last snapshot of each simulation. Color of ATP is changing from red to white and white to blue along the time course.

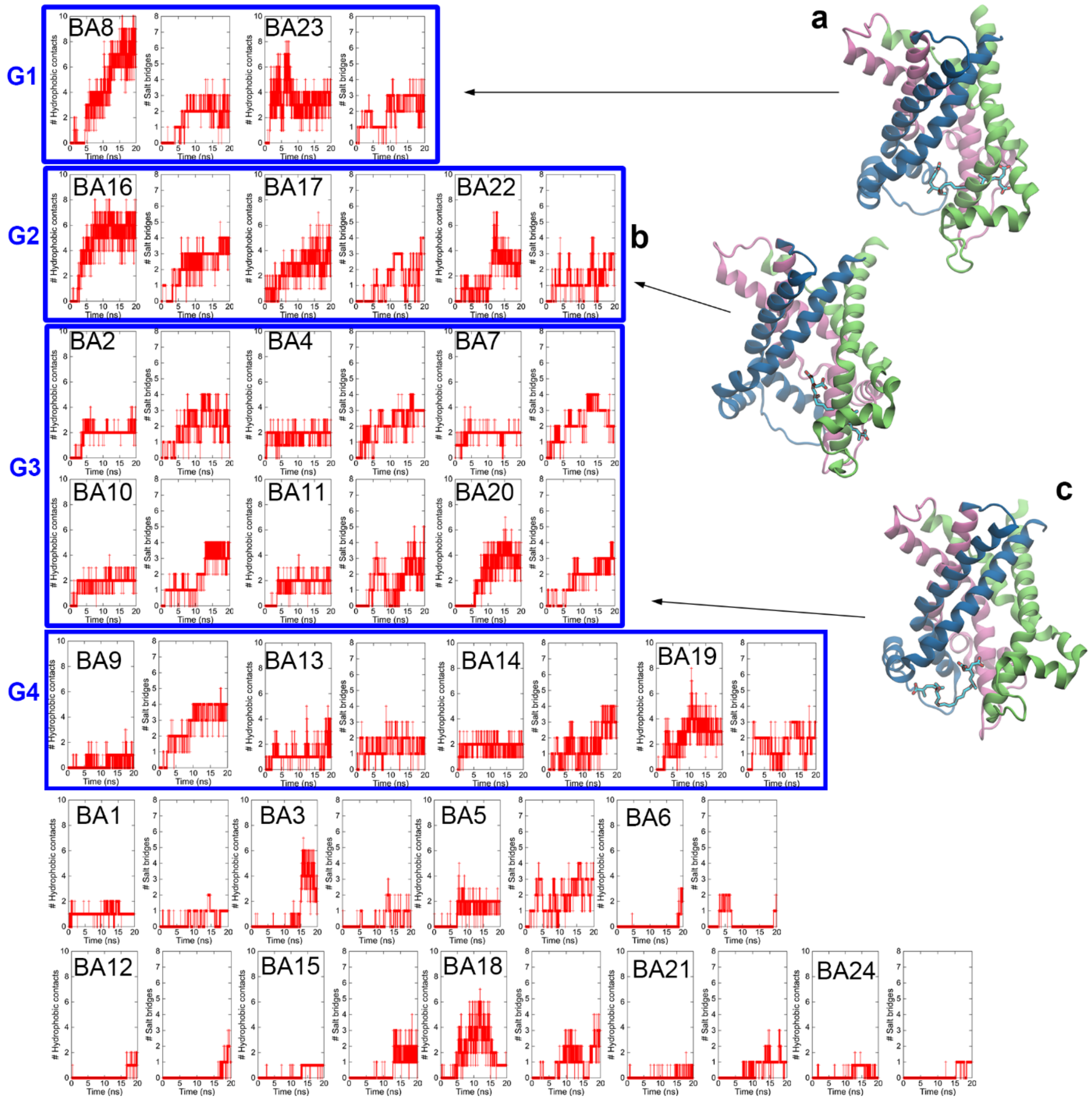

Fig I

Binding of BA to the matrix pore of the IF form. Time courses of the numbers of hydrophobic contacts (left) and salt-bridges (right) in BA binding simulations (BA1 and BA24) are shown. The binding structures of the IF form were characterized by binding poses, and accordingly classified into 4 classes, G1-4. Bottom panels which are not classified into G1-4 indicate the simulations where binding of BA was absent. (*a*) In two structures in G1, the carboxylate at MCT formed salt-bridges with basic residues in the central pore and those at DCT did with basic residues located in crack I-II, respectively. (*b*) In three structures in G2, the carboxylates at MCT and DCT interacted with basic resides located in crack I-II and the central pore, respectively. (*c*) BA in six structures in G3 interacted with residues in RRRMMM motif located at the C-terminal end of TM5 which is strictly conserved among AAC orthologues (32). (*d*) Four unclearly characterized binding structures are classified into G4.

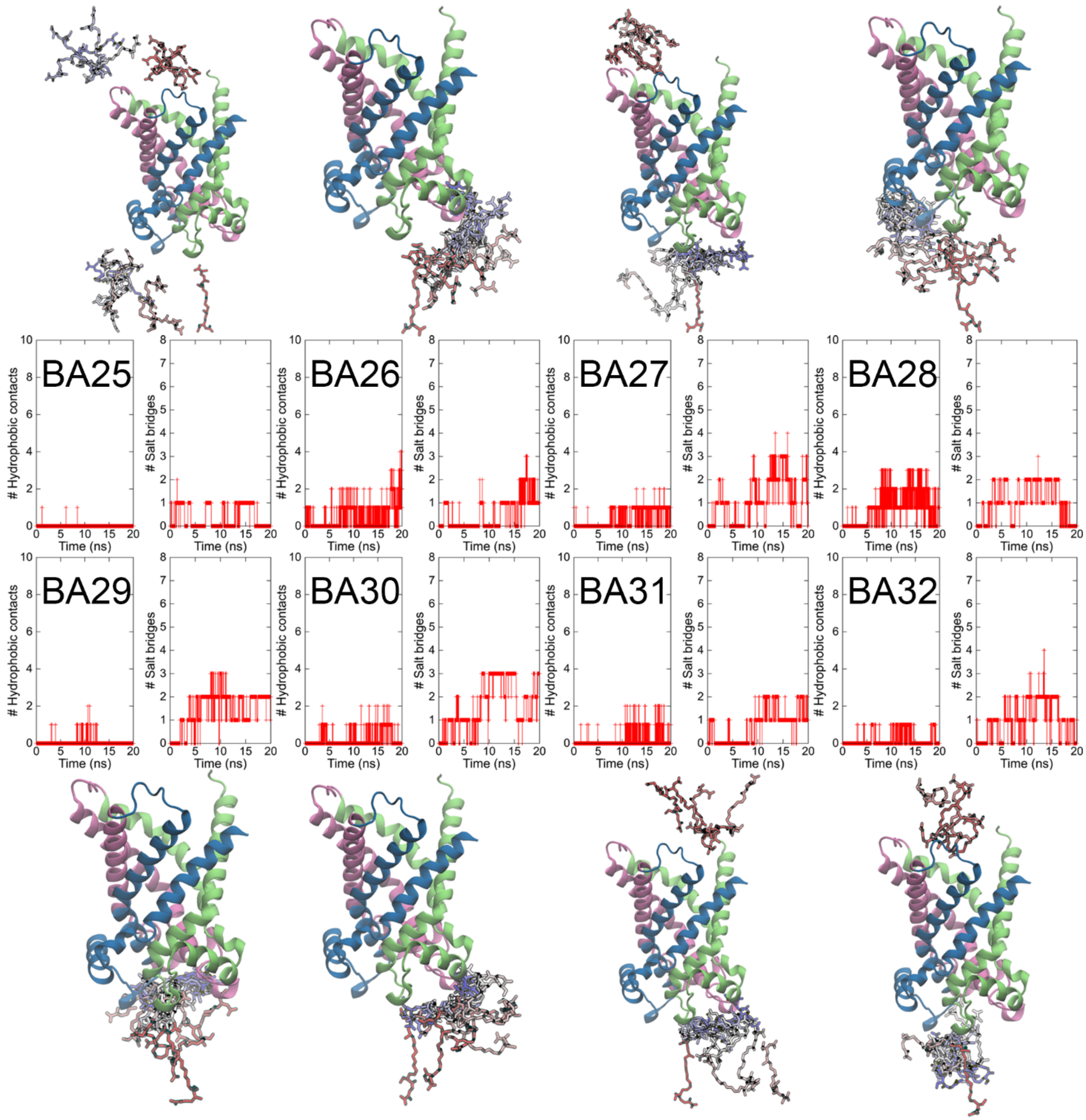

Fig J

Binding simulation of BA to the OF form (BA25–32). Time courses of the number of hydrophobic contacts (left) and salt-bridges (right) are shown. Trajectories of BA at every 1 ns are mapped onto the X-ray structure. Color of BA is changing from red to white and white to blue along the time course.

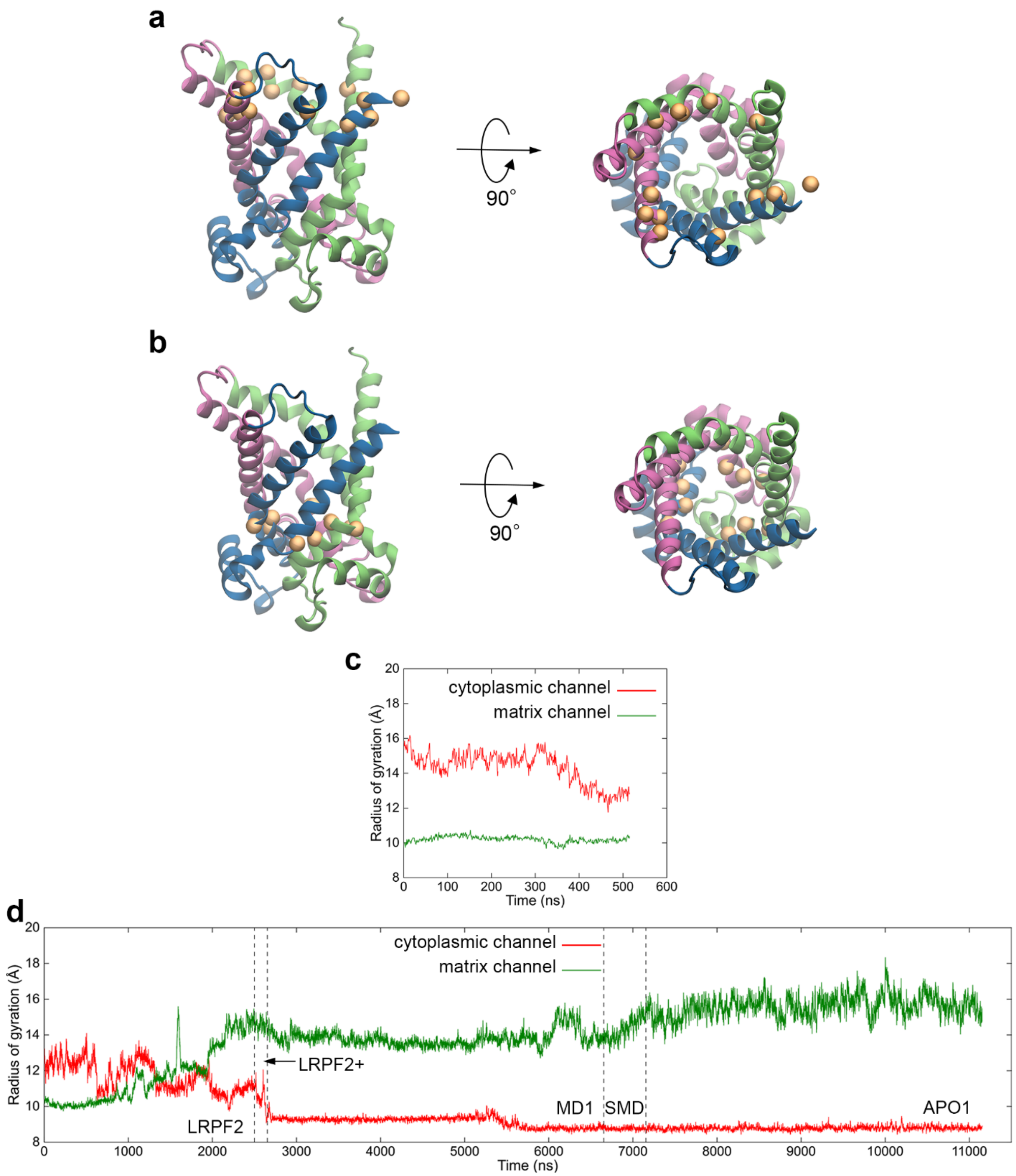

Fig K

Closure of the cytoplasmic channel and opening of the matrix one expressed with radii of gyration. (*a, b*) C_{α}-atoms of residues for which radii of gyration of the cytoplasmic channel (*a*) and the matrix one (*b*) were evaluated [ref. 42] are indicated with spheres in the crystallographic structure (PDB ID: 1OKC). See **S1 Text** for detailed information on the radii of gyration. (*c, d*) Time courses of the radii of gyration in ADP-binding (*c*) and LRPF (*d*) simulations.

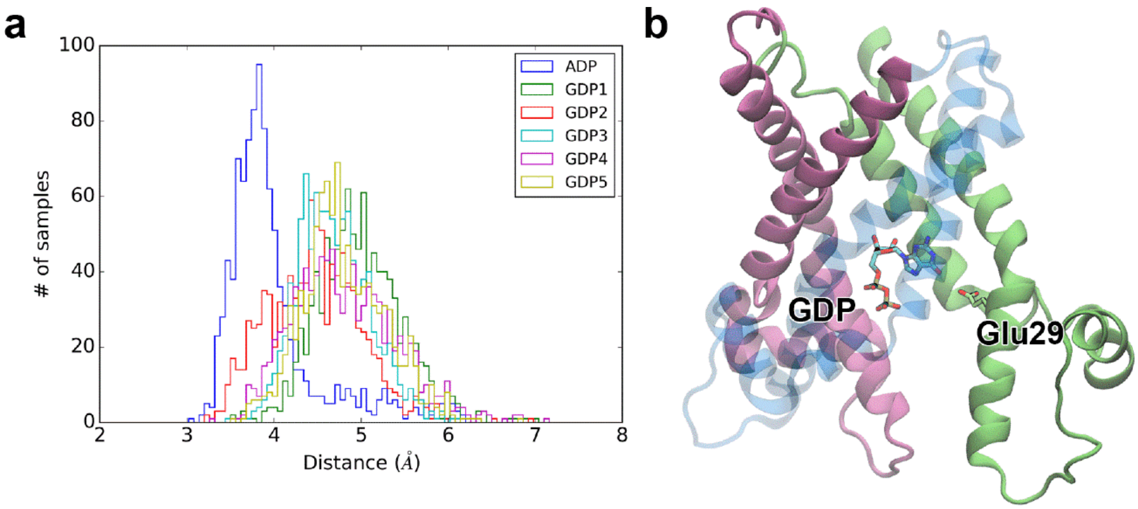

Fig L

Equilibrium simulations of nucleotide-bound form of AAC. (*a*) Nearest distance distributions calculated from 10-ns unbiased simulations (1000 samples). Nearest distance between C_δ atom of Glu29 and heavy atom of nucleotide (ADP or GDP) was measured. Distribution corresponding to ADP-bound form was calculated from 2000–2100 ns segment of LRPF2 simulation. (*b*) A snapshot illustrating GDP-bound form of AAC.
